# Full Restoration of *Brucella*-Infected Dendritic Cell Functionality through Vγ9Vδ2 T Helper Type 1 Crosstalk

**DOI:** 10.1371/journal.pone.0043613

**Published:** 2012-08-22

**Authors:** Ming Ni, Delphine Martire, Emmanuel Scotet, Marc Bonneville, Francoise Sanchez, Virginie Lafont

**Affiliations:** 1 Université Montpellier 1, Centre d’études d’agents Pathogènes et Biotechnologies pour la Santé (CPBS) Montpellier, France; 2 CNRS, UMR 5236, CPBS, F-34965 Montpellier, France; 3 Université Montpellier 2, CPBS, F-34095 Montpellier, France; 4 Inserm Unité Mixte de Recherche 892, Centre de Recherche en Cancérologie Nantes-Angers, Institut de Recherche Thérapeutique Université de Nantes, Nantes, France; University of Palermo, Italy

## Abstract

Vγ9Vδ2 T cells play an important role in the immune response to infectious agents but the mechanisms contributing to this immune process remain to be better characterized. Following their activation, Vγ9Vδ2 T cells develop cytotoxic activity against infected cells, secrete large amounts of cytokines and influence the function of other effectors of immunity, notably cells playing a key role in the initiation of the adaptive immune response such as dendritic cells. *Brucella* infection dramatically impairs dendritic cell maturation and their capacity to present antigens to T cells. Herein, we investigated whether V T cells have the ability to restore the full functional capacities of *Brucella*-infected dendritic cells. Using an in vitro multicellular infection model, we showed that: 1/*Brucella*-infected dendritic cells activate Vγ9Vδ2 T cells through contact-dependent mechanisms, 2/activated Vγ9Vδ2 T cells induce full differentiation into IL-12 producing cells of *Brucella*-infected dendritic cells with functional antigen presentation activity. Furthermore, phosphoantigen-activated Vγ9Vδ2 T cells also play a role in triggering the maturation process of dendritic cells already infected for 24 h. This suggests that activated Vγ9Vδ2 T cells could be used to modulate the outcome of infectious diseases by promoting an adjuvant effect in dendritic cell-based cellular therapies.

## Introduction

In humans, T cells expressing Vγ9Vδ2 antigen receptors belong to the family of innate lymphocytes. Due to their particular properties, innate lymphocytes act as a bridge between innate and adaptive immunity and play a crucial role in both antitumoral and anti-infectious responses. Vγ9Vδ2 T cells are activated through the T cell receptor (TCR) by phosphorylated non-peptidic antigens called phosphoantigens [1], [2], [3] that have been isolated from intracellular pathogens as metabolites involved in the pathway of isoprenoid synthesis [4]. Phosphoantigen recognition does not require antigen processing or presentation by major histocompatibility complex molecules [5], [6]. Also, pharmacological agents (aminobiphosphonates, statins) acting on isoprenoid pathway have been shown to modulate Vγ9Vδ2 T cell activity [7], [8], [9]. More recently, complexes comprising ATP synthase subunits and expressed on the surface of some tumor cells have also been described to activate Vγ9Vδ2 T cells [10]. Due to their activation property and broad reactivity, Vγ9Vδ2 T cells have a potential interest as therapeutic targets. Indeed, activated Vγ9Vδ2 T cells have many effects in the immune response: they display rapid cytotoxic activity against infected or tumoral cells, and release an array of chemokines and cytokines that contribute to neutrophil recruitment and activation, monocyte differentiation, B cell-maturation into-antibody-producing plasma cells and dendritic cell (DC) maturation into antigen-presenting cells (APCs) (reviewed in [11]). DCs represent the most important APCs exhibiting the unique capacity to initiate primary T cell responses and then play a crucial role in the anti-infectious immune response [12]. During an infection, pathogen recognition by TLRs induces DC maturation and this process is crucial for the ability of DCs to initiate adaptive immune responses. However, several intracellular bacterial pathogens interfere with their maturation mechanisms and thus perturb the initiation of adaptive immune response (example include *Mycobacterium* tuberculosis, *Brucella* suis, *Coxiella* burnetii) [13], [14], [15], [16]. Recent studies have shown that in addition to TLR recruitment, DCs can also interact and cross-talk with innate lymphocytes leading to innate lymphocyte activation and DC maturation. The interaction between phosphoantigen-activated Vγ9Vδ2 T cells and DCs triggers their maturation [17], [18], [19]. This described mechanism might help DCs to avoid the maturation inhibition induced by pathogens. Accordingly several molecules (aminobiphosphonates, synthetic phosphoantigens) with Vγ9Vδ2 T cell-activating properties which are being tested in other disorders (anti-tumoral treatment) could also be used in anti-infectious therapies [20], [21], [22]. The final outcome of these cellular interactions may have a dramatic impact on the quality and strength of the downstream immune responses, mainly in the context of early responses to tumour cells and infectious agents.

*Brucella* is a facultative intracellular bacterium responsible for a disease in animals and humans. Brucellosis is one of the five most common bacterial zoonoses in the world and the most prevalent anthropozoonosis with more than 500000 new cases annually [23], [24]. Also known as Malta fever, human brucellosis consists in acute infection, characterized by undulant fever and asthenia, which evolves in 30% of non-treated infected patients into a chronic disease with erratic recurrent fevers and localized infections such as endocarditis, encephalitis, spondylitis. Human infections occur through inhalation of aerosols or consumption of infected food. Following invasion of the lymphoid system, the bacteria develop within mononuclear phagocytes, and infected cells could participate in the dissemination of the bacteria in specific locations of the body. More recent reports have shown that *Brucella* also infect DCs and abrogates their maturation process, IL-12 production and antigen-presenting activity [13]. We previously showed that Vγ9Vδ2 T cells could inhibit intracellular *Brucella* growth and development through a combination of mechanisms: (i) cytotoxicity [25], (ii) macrophage activation and bactericidal activity through cytokine and chemokine secretion [25], (iii) anti-bacterial effects via granulysin [25] and LL-37 [26].

In this study we investigated whether Vγ9Vδ2 T cells could be used as a new therapeutic approach to modulate *Brucella*-infected DC activity. Using a multicellular infection model, we showed that Vγ9Vδ2 T cell activation was induced by *Brucella*-infected DCs and required a cell-cell contact. Reciprocally, the presence of activated Vγ9Vδ2 T cells mediated full maturation of *Brucella*-infected DCs into IL-12 producting cells. IFN-γ and TNF-α produced by activated Vγ9Vδ2 T cells contributed to the maturation process. We also studied whether mature infected DCs could recover proper APC function and ability to efficiently trigger proliferation of naive T cells. Furthermore, we demonstrated that full functional APC activity of *Brucella*-infected DCs can be restored by Vγ9Vδ2 T cells even 24 h post-infection (p.i.). This study provides the first evidence for Vγ9Vδ2 T cell ability to modulate DC activity in the context of infections by pathogens which totally impair infected-DC maturation. Overall, these results indicate that Vγ9Vδ2 T cell might promote an adjuvant effect in the DC-based cellular therapies and thus modulate the balance of the adaptive response and influence the outcome of infectious diseases.

## Materials and Methods

### Antigens, Antibodies and Reagents

HMB-PP ((E) -4 hydroxy -3 methyl –but -2 enyl –pyrophosphate) was a generous gift from J.L. Montero (Institut des Biomolecules Max Mousseron, CNRS UMR 5247, Montpellier, France). Anti-human and isotypically matched control mouse Abs (conjugated or not) were all purchased from BD Biosciences (San Jose, CA, USA): anti-CD1a (clone Hi149), anti-CD3 (clone UCHT1), anti-CD83 (clone HB15e), anti-CD86 (clone 2331 FUN-1), anti-IFN-γ (clones B27 and NIB42), anti-TNF-α (clone MAb1) and anti-γ9 TCR (clone B3) mAbs. Recombinant human GM-CSF and IL-4 from ImmunoTools (Friesoyte, Germany) were used at a concentration of 15 ng/ml and 10 ng/ml respectively. Recombinant human IL-2 (rhIL-2) was from Chiron, (Emeryville, CA, USA).

### Ethics

Anonymous blood donations were obtained from French Establishment of Blood (EFS).

### Cells

PBMC from healthy donors were prepared by density centrifugation on Ficoll-Paque (Eurobio, Les Ulis, France). CD14+ monocytes were purified from PBMC by magnetic positive separation (Miltenyi Biotec, Paris, France). Vγ9Vδ2 T cells were purified from the remaining cells using an anti-γ9 mAb and goat anti-mouse IgG-coated magnetic beads (Dynal, Compiégne, France) according to the manufacturer’s instructions. After detachment from the beads, Vγ9Vδ2 T cells were stimulated with HMB-PP (10 nM) in the presence of autologous monocytes and rhIL-2 (20 ng/ml). Vγ9Vδ2 T cells were expanded in complete medium (RPMI 1640/glutamax, Life Technologies, Paisley, UK) supplemented with 5% FCS, 5% heat-inactivated- human AB serum and rhIL-2. After 3 weeks of culture, γδ T cells were >98% CD3^+^Vγ9^+^Vδ2^+^ as assessed by FACS analysis. Monocytes (0.7×10^6^ cells/ml) were further differentiated into immature DCs in complete culture medium (RPMI 1640/glutamax supplemented with 10% heat-inactivated FCS in the presence of GM-CSF (15 ng/ml) and IL-4 (10 ng/ml) for 6 days. Fresh medium plus cytokines were added every 2 days and cell cultures contained >90% of immature DCs (CD1a+ and CD14- expression) as assessed by flow cytometry analysis at day 6.

### Infection of DCs with *Brucella suis*

Immature DCs (3.5×10^6^ cells/ml) were infected with *Brucella suis (B. suis)* 1330 at the following multiplicities of infection (MOI, 2, 5, 20 and 50), otherwise when it is not mentioned MOI used is 20. After 1 h, DCs were washed and resuspended (0.7×10^6^ cells/ml) alone or in the presence of autologous Vγ9Vδ2 T cells activated or not by HMB-PP (0.2 nM) with a DCs/Vγ9Vδ2 T cells ratio of 1∶1 in complete culture medium. Gentamicin was added in culture medium to kill non-phagocytosed bacteria and avoided extracellular development of *Brucella*. Although slower than *E. coli*, the growth of *Brucella suis* is fast enough to invade in 24 h the culture medium and perturb cell culture. To evaluate cell-cell contact requirement, a transwell system was used (0.4 µm, Millipore, Bedford, MA). When mentioned, blocking mAbs to TNF-α (15 µg/ml) or IFN-γ (25 µg/ml) were added in the wells. In some experiments, Vγ9Vδ2 T cells were only added to DCs 24 h post infection (p.i.). For all conditions and times, supernatants were collected to assay cytokines and cells were harvested for staining analysis. When not mentioned in the text and figures, coincubation experiments were performed with 3 week-expanded γδ T cells.

### Maturation Marker Analysis

At different time points p.i., cells were harvested and incubated with FITC-conjugated mAbs to CD83, CD86 or their isotype-matched controls for 30 min at 4°C, then washed, fixed and analyzed on FACSCalibur (Becton Dickinson) using CellQuest software.

### Intracellular Staining of IFN-γ

To study intracellular IFN-γ, cocultures were realized in the presence of brefeldin A (1 µl/ml; BD Biosciences) for the last 5 h. At 24 h and 48 h p.i., cells were harvested, stained with a PE-conjugated anti-CD3 mAb, fixed, and permeablized for 20 min at 4°C (BD Cytofix/Cytoperm™ Fixation/Permeabilization kit) according to the manufacturer’s instructions. Then, cells were incubated with Alexa Fluo 488-conjugated anti-IFN-γ or their isotype-matched controls for 30 min at 4°C in the dark, washed and analyzed on FACSCalibur using CellQuest software.

### Cytokine Measurement

Supernatants from infection experiments were collected at various times p.i. and assayed for IFN-γ, IL-12 using specific OptEIA human enzyme-linked immunosorbent assay sets (BD Pharmingen) according to the manufacturer’s instructions.

**Figure 1:**
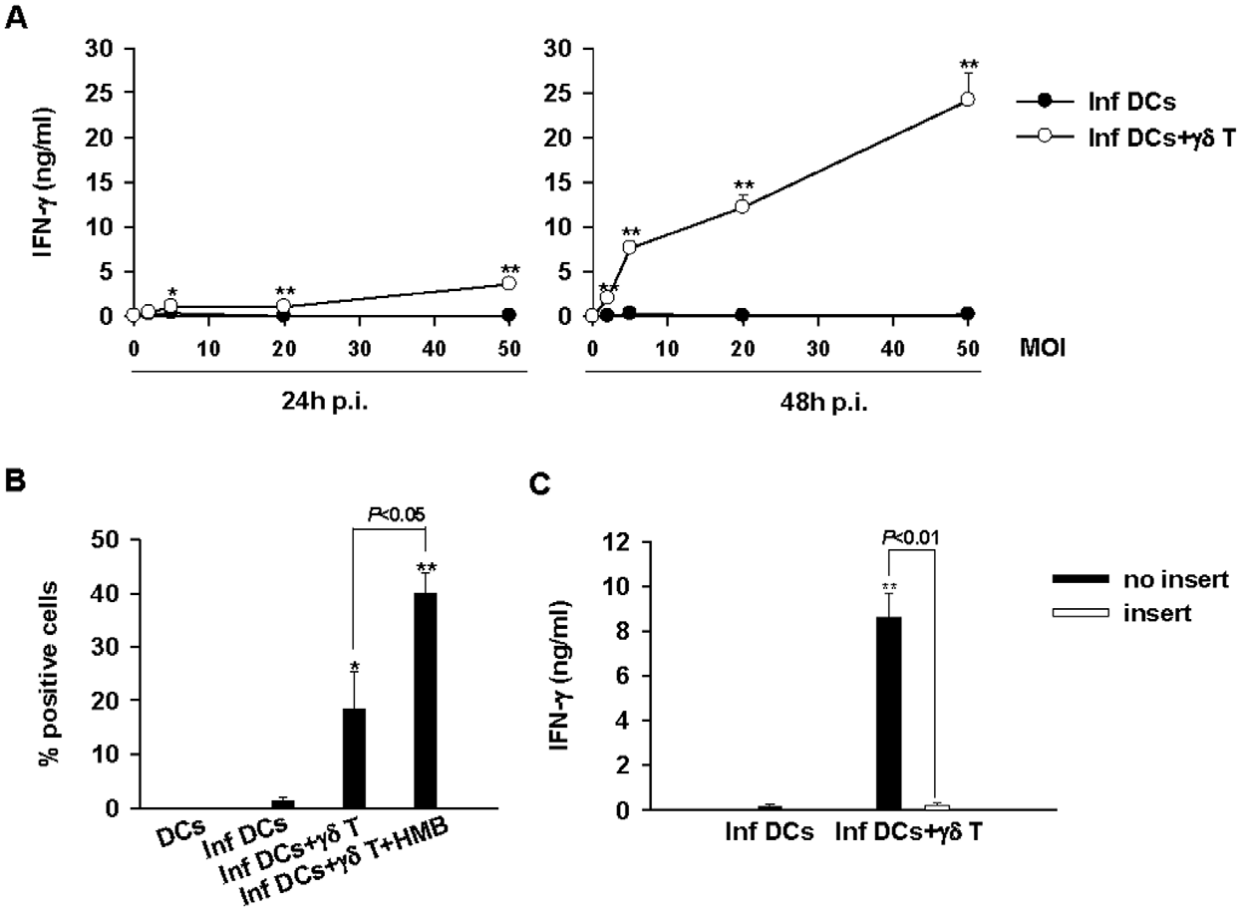
IFN-γ production by Vγ9Vδ2 T cells is induced by *Brucella*-infected DCs and required cell-cell contact. A/ Correlation between the MOI used to infected DCs and IFNγ production by Vγ9Vδ2 T cells. DCs infected or not with different MOI (2, 5, 20, and 50) were cultured alone or in the presence of Vγ9Vδ2 T cells. At 24 h and 48 h p.i., supernatants were collected and the concentration of IFN-γ was assayed by ELISA test. A significant difference between infected DCs in the presence or not of Vγ9Vδ2 T cells was calculated by using a Student’s *t* test and is indicated by (*) where p<0.05 and (**) where p<0.01. Data shown are the mean +/− SD of triplicates and are representative of three experiments performed with cells from different donors. B/ Intracellular accumulation of IFN-γ in Vγ9Vδ2 T cells. Untreated or stimulated (0.2 nM HMB-PP) Vγ9Vγ2 T cells were cocultured with non- or *Brucella*-infected DCs (MOI = 20). At 24 h p.i., intracellular IFN-γ was analyzed in Vγ9Vδ2 T cells by flow cytometry. The Student’s *t* test was used to calculate significant differences between: 1- infected DCs in the presence or not of Vγ9Vδ2 T cells and were indicated by (*) where *p*<0.05 and (**) where *p*<0.01 and 2- untreated or HMB-stimulated Vγ9Vδ2 T cells and were directly indicated on the graph. Data shown are the mean +/− SD of three independent experiments. C/ IFN-γ production requires cell-cell contacts between infected DCs and Vγ9Vδ2 T cells. *Brucella*-infected DCs (MOI = 20) were cocultured with Vγ9Vδ2 T cells in contact or separated by a trans-well chamber system (insert). At 48 h p.i., supernatants were collected and IFN-γ was measured by ELISA. The Student’s *t* test was used to calculate significant differences between: 1- infected DCs in the presence or not of Vγ9Vδ2 T cells and was indicated by (**) where p<0.01 and 2- Vγ9Vδ2 T cells in contact or not with infected DCs was directly indicated on the graph. Data shown are the mean +/− SD of triplicates and are representative of three independent experiments.

### Antigen Presentation to Naive Human T Lymphocytes

Human naive CD4+ T cells (CD3^+^CD4^+^CD45RA^+^) were isolated by magnetic depletion of memory CD4+ T cells and non-CD4+ T cells with the naive CD4+ T cell isolation kit II (Miltenyi Biotec) according to the manufacturer’s instructions. Naive CD4^+^ CD45RA^+^ T cells with a purity >97% were stained with 2.5 µM CFSE (Sigma) at 37°C for 10 min, then extensively washed, and plated in a 96-well culture plate (10^5^ cells/well). *B. suis*-, *E. coli*- or not infected DCs were cultured with or without Vγ9Vδ2 T cells for 24 h and added to naive CD4+ T cells at various DCs/T cells ratio range from 0 to 0.1. Five days later, cells were harvested and CFSE fluorescence was analyzed by flow cytometry using a FACSCalibur cytometer.

### Statistical Analysis

Wilcoxon rank tests, un-paired or paired Student’s t-tests were applied to determine statistical differences in T cell proliferation assays, cytokine measurements and maturation marker expression analysis respectively. A statistical difference was considered significant when p<0.05.

## Results

### *Brucella*-infected DCs Trigger IFN-γ Production in Vγ9Vδ2 T Cells through Cell-cell Contact Mechanisms

We previously reported that *Brucella*-infected macrophages were able to activate Vγ9Vδ2 T cells through a TCR-dependent mechanism [25], [27]. More recently, Billard et al. have shown that *Brucella suis* infects, proliferates and survives in human DCs as well or even better than in macrophages [28]. Herein, we investigated the impact of DC infection on the activity of Vγ9Vδ2 T cells assessed by the production of IFN-γ. Using the co-culture model of infection described in materials and methods, we showed that Vγ9Vδ2 T cells produce IFN-γ when they are cultured in the presence of the phosphoantigen HMB-PP and/or *Brucella*-infected DCs (Fig. 1 A and B). Vγ9Vδ2 T cells alone (data not shown) or cultured in the presence of non-infected DCs (Fig. 1A) did not produce IFN-γ. Moreover, the amounts of IFN-γ produced correlated with the number of bacteria used to infect DCs (MOI) and were maximal at MOI of 50 at 48 h p.i. (Fig. 1A).

**Figure 2:**
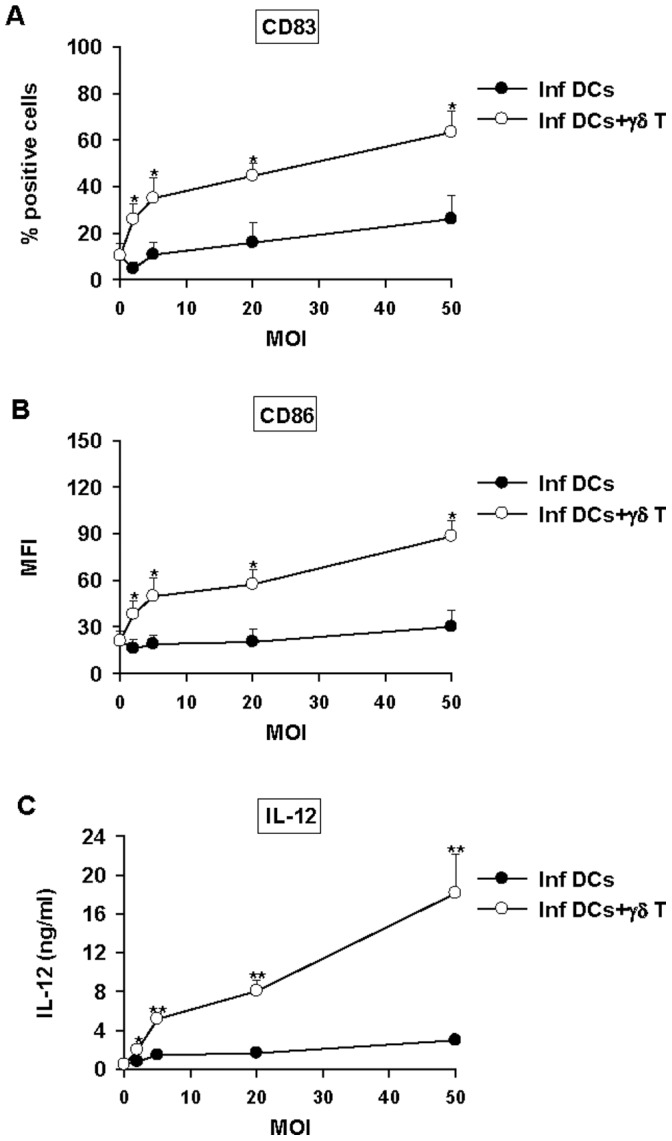
Vγ9Vδ2 T cells induce full maturation of *Brucella*-infected DCs. Non- or *Brucella*-infected DCs (MOI = 2, 5, 20, and 50) were cocultured with Vγ9Vδ2 T cells. At 48 h p.i., supernatants were collected and cells were stained with FITC-conjugated mAbs to CD83 (A), CD86 (B). CD83 and CD86 expression analyses were realized on CD1a+ cells by flow cytometry and the results were expressed as percentage of positive cells for CD83 and mean fluorescence intensity (MFI) for CD86. Data shown are the mean +/− SD of three independent experiments performed with cells from different donors. IL-12 was assessed by ELISA test in the collected supernatants (C). Data shown are the mean +/− SD of triplicates and are representative of three experiments. A significant difference between infected DCs in the presence or not of Vγ9Vδ2 T cells was calculated by using Student’s t test and is indicated by (*) where p<0.05 and (**) where p<0.01.

To assess the respective implication of soluble factors or contact-dependent mechanisms in IFN-γ production, we used a two chamber system where *Brucella*-infected DCs are separated from Vγ9Vδ2 T cells by a semi-permeable membrane through which only soluble factors can cross. When Vγ9Vδ2 T cells were not in contact with infected DCs, IFN-γ production is abrogated (Fig. 1C). Therefore, IFN-γ production and Vγ9Vδ2 T cell activation require cell-cell contact with *Brucella*-infected DCs.

**Figure 3:**
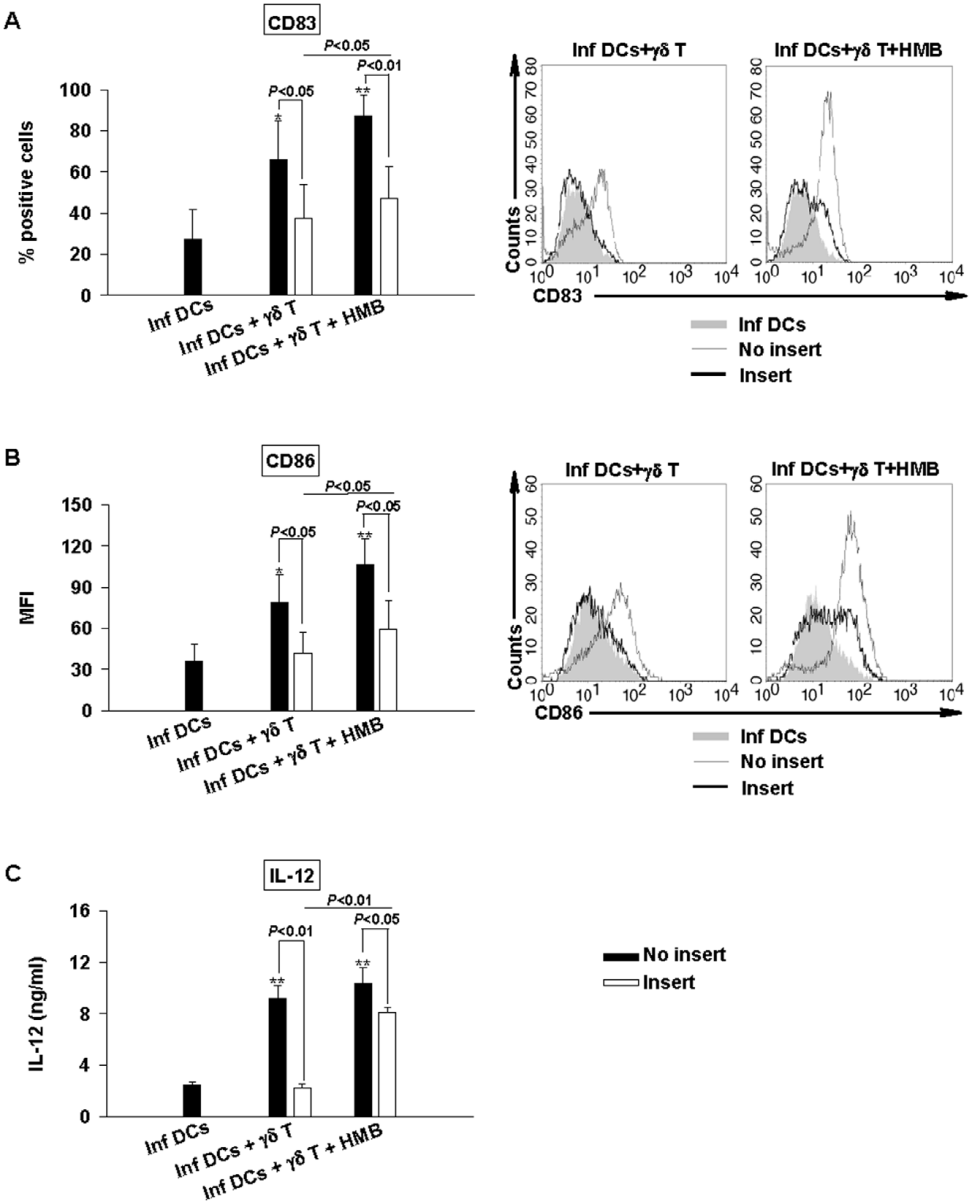
Cell-cell contacts between Vγ9Vδ2 T cells and infected DCs are required for maturation process. *Brucella*-infected DCs (MOI = 20) were cocultured in contact (black bars) or separated by a trans-well chambers (insert, white bars) with Vγ9Vδ2 T cells in the presence or not of HMB-PP (0.2 nM). At 48 h p.i., supernatants were collected and cells were stained. CD83 (A, left panel) and CD86 (B, left panel) expression analyses were realized on CD1a+ cells by flow cytometry. Data shown are the mean +/− SD of three independent experiments performed with cells from different donors. Representative flow cytometry histograms show the expression of CD83 (A, right panel) and CD86 (B, right panel) of infected DCs alone (filled grey), cocultured in contact (grey line) or separated (black line) with HMB- or not- stimulated Vγ9Vδ2 T cells. IL-12 was assessed by ELISA test in the collected supernatants (C). Data shown are the mean +/− SD of triplicates and are representative of the three experiments. A significant difference between infected DCs in the presence or not of Vγ9Vδ2 T cells was calculated by using Student’s t test and is indicated by (*) where p<0.05 and (**) where p<0.01. Significant differences between infected DCs in contact or not with Vγ9Vδ2 T cells and between HMB- or not- stimulated Vγ9Vδ2 T cells are indicated directly on the graph.

### 2Vγ9Vδ2 T Cells Mediated Full Maturation of *Brucella*-infected DCs

Several studies have reported the ability of activated Vγ9Vδ2 T cells to induce the maturation of iDCs [17], [18], [19]. However, no study with DCs infected by intracellular pathogens which totally abrogate or avoid the maturation process such as *Brucella* has been performed so far. In these conditions, infection by *Brucella* represents a good model to assess the ability of Vγ9Vδ2 T cells to induce maturation of infected DCs. DCs were infected with different MOIs and then cultured alone or in the presence of Vγ9Vδ2 T cells. At 48 h p.i., we analyzed DCs for the expression of maturation markers CD83 and CD86 (Fig. 2A, 2B and S1). Co-culture of *Brucella*-infected DCs with Vγ9Vδ2 T cells upregulated the expression of the costimulatory molecules CD83 and CD86 (Fig. 2A and B, S1). Moreover, CD83 and CD86 upregulation correlated with both MOI used to infect DCs and Vγ9Vδ2 T cell activation state (as measured by IFN-γ production) and reached levels similar to those with DCs stimulated by classical stimuli, such as *E. coli* LPS (Fig. S2).

To investigate whether the phenotypic changes in *Brucella*-infected DCs paralleled modification of their functional properties, we assessed IL-12 production in infection experiments (Fig. 2C). As already reported, we confirmed that *Brucella* abrogates IL-12 production by infected DCs. By contrast, strong IL-12 production was observed in *Brucella*-infected DCs cocultured with Vγ9Vδ2 T cells (Fig. 2C). IL-12 production correlated with the MOIs and reached the maximum for a MOI of 50. To be more relevant to the *in vivo* situation, we have also performed these experiments with fresh purified γδ T cells and found similar data. Primary Vγ9Vδ2 T cells are able to induce *Brucella*-infected DC maturation and IL-12 production similarly than long-term expanded γδ T cells (Fig. S3). Overall, we concluded that the presence of Vγ9Vδ2 T cells mediated full maturation of *Brucella*-infected DCs and their differentiation in IL-12 producing cells.

### Role of Contact-dependent Mechanisms Versus Soluble Factors in *Brucella*-infected DC Maturation and IL-12 Production

To investigate the role played by soluble factors *vs* cell-cell contact mechanisms in the maturation of *Brucella*-infected DCs induced by Vγ9Vδ2 T cells, infection experiments were performed using a two chamber system (Fig. 3). When Vγ9Vγ2 T cells were not in contact with *Brucella*-infected DCs, both the maturation process and IL-12 production were abrogated. This is consistent with the above results in which we demonstrated that Vγ9Vγ2 T cell activation requires contact-dependent mechanisms with infected DCs leading to reciprocal effect on DC maturation. However, when Vγ9Vδ2 T cells are separated from DCs but activated by the addition of exogenous phosphoantigens (HMB-PP), there was a partial restoration of DC maturation with a weak upregulation of maturation markers CD83 and CD86 and a production of IL-12. These indicate that complete DC maturation in IL-12 producing cells requires a combination of both contact-dependent mechanisms and soluble factors.

### Differential Contribution of TNF-α and IFN-γ to *Brucella*-infected DC Maturation and IL-12 Production

**Figure 4:**
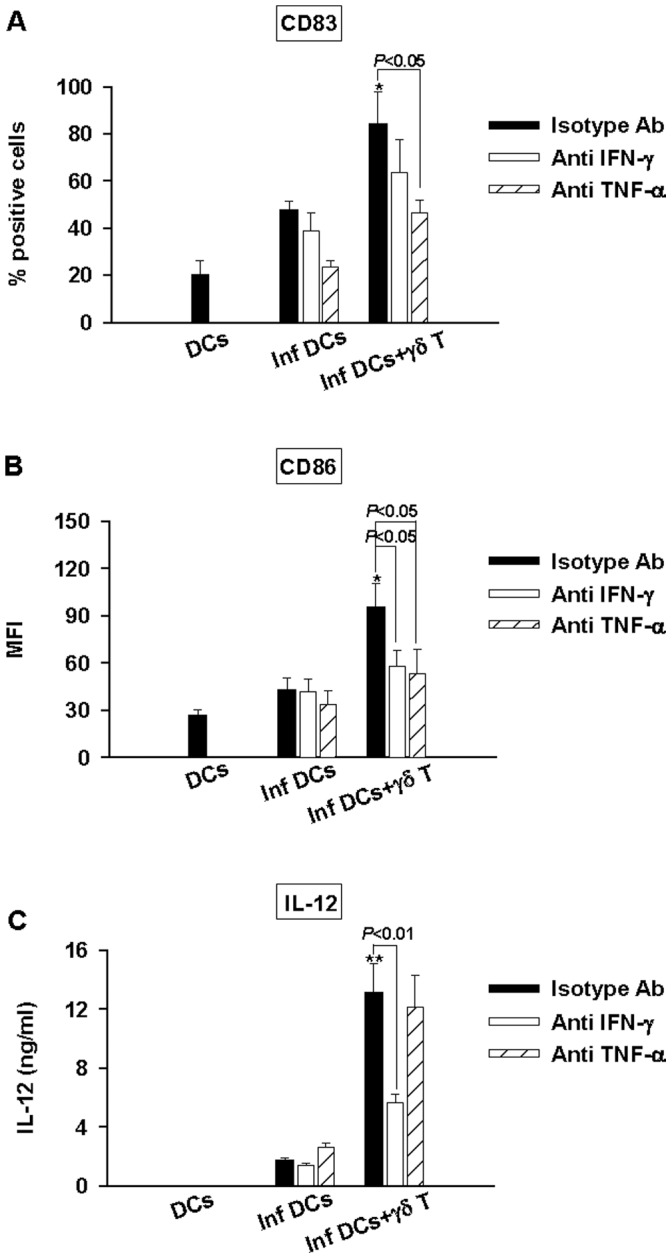
Role of IFN-γ and TNF-α on DC maturation induced by Vγ9Vδ2 T cells. DCs (DC), infected DCs (inf DC) and infected DCs in the presence of Vγ9Vδ2 T cells (infDCs+γδT) were cultured with neutralizing mAbs to IFN-γ (white bars), TNF-α (striped white bars) or with isotype control Ab (black bars). At 48 h p.i., supernatants were collected and cells were stained with FITC-conjugated mAbs to CD83 (A), CD86 (B). CD83 and CD86 expression analysis were realized on CD1a+ cells by flow cytometry. IL-12 was assessed by ELISA in the collected supernatants (C). Data shown are the mean +/− SD of triplicates and are representative of the three experiments. The Student’s *t* test was used to calculate significant differences between: - 1: infected DCs in the presence or not of Vγ9Vδ2 T cells and was indicated by (*) where *p*<0. 05 and (**) where *p*<0.01 and 2: infected DCs cocultured with Vγ9Vδ2 T cells in the presence of neutralizing mAb or isotype control and is directly indicated on the graph.

Phosphoantigen-activated Vγ9Vδ2 T cells mainly produce high amounts of TNF-α and IFN-γ [29]. These two cytokines are involved in DC maturation and IL-12 production in non-infected DCs [18]. To further determine the role of these cytokines on the impact of Vγ9Vδ2 T cells in *Brucella*-infected DCs, we performed infection experiments in the presence of blocking anti-TNF-α or anti-IFN-γ mAbs (Fig. 4). While anti-TNF-α mAb similarly blocked CD83 and CD86 upregulation, anti-IFN-γ mAb had more effect on the impairment of CD86 expression (Fig. 4A and B). Inversely, anti-TNF-α mAb had a weak or no effect on IL-12 production whereas anti-IFN-γ mAb was more efficient (Fig. 4C). Moreover, no additional effect was observed when Vγ9Vδ2 cells were cultured in the presence of both anti-IFN-γ and anti-TNF-α mAbs (data not shown). Taken together, these results suggest that IFN-γ and TNF-α are differently involved in *Brucella*-infected DC maturation process and IL-12 production.

### *Brucella*-infected DCs Cultured in the Presence of Vγ9Vδ2 T Cells Induce Proliferation of Naive CD4+ T Cells

**Figure 5:**
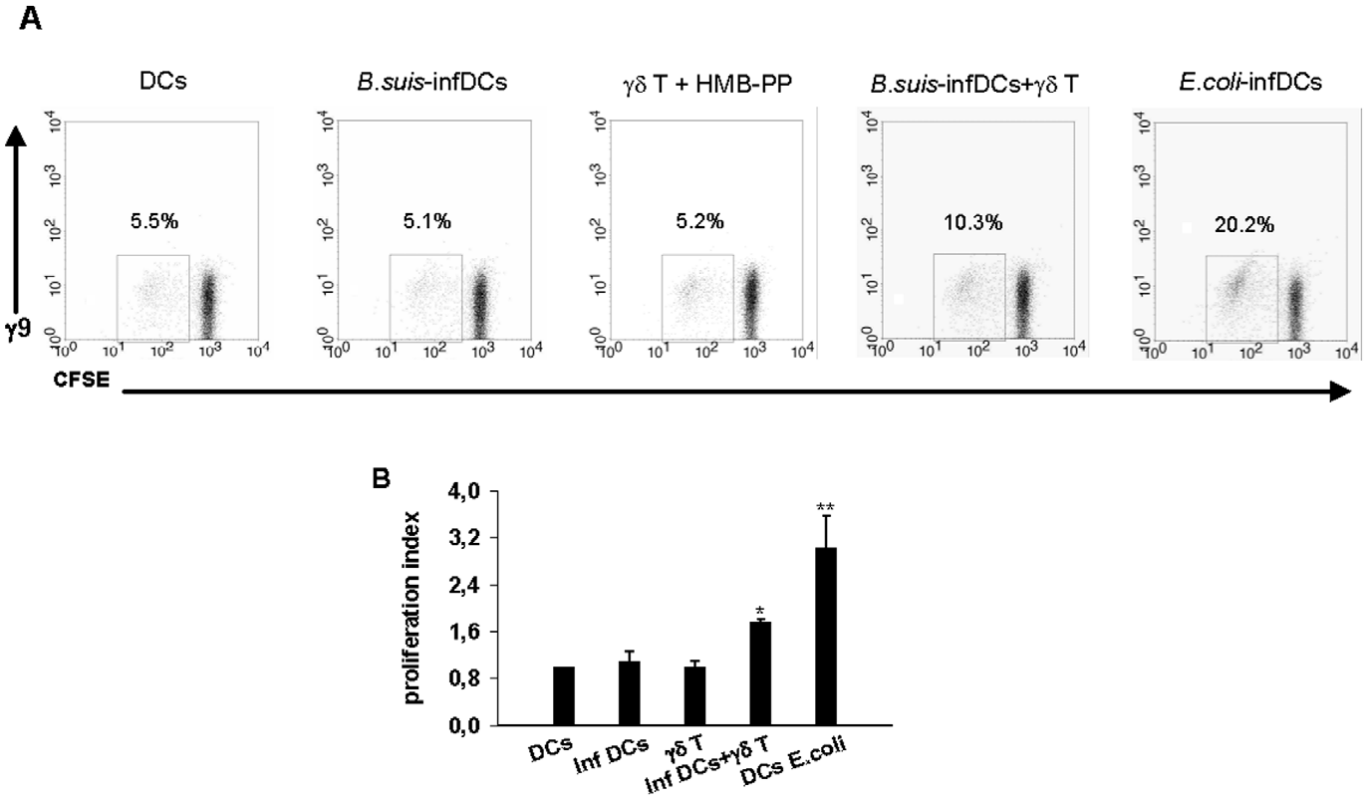
Vγ9Vδ2 T cells increase the capacity of *Brucella*-infected DCs to induce naive T cell proliferation. *B.suis*- or *E. coli-*infected DCs (MOI = 20) and Vγ9Vδ2 T cells were cultured alone or in coculture for 24 h then CFSE-stained naive CD4+ T cells were added with DC/T cell ratio: 0.1. After 5 days, the proliferation of CD4+ T cells (γ9- CFSE+) was analyzed by flow cytometry and the proliferation index was calculated by comparison with non-infected cells. (A) Representative flow cytometry dot plots showing the proliferation of CD4+ T cell. Values for the percentages of proliferated CFSE+ and γδ− cells are indicated upper of the region. (B) Data represent the mean of duplicates +/− SD of three independent experiments performed with cells from different donors. Statistical differences were calculated by comparison with *Brucella*-infected DCs by using Wilcoxon Rank test and are indicated by (*) where p<0. 05 and (**) where p<0.01.

To investigate whether maturation of *Brucella*-infected DCs into IL-12-producing cells induced by Vγ9Vδ2 T cells also leads to functional T cell priming activity, we assessed the ability of *Brucella*-infected DCs cocultured with Vγ9Vδ2 T cells to stimulate the proliferation of naive CD4+ CD45RA+T lymphocytes. *Brucella*-infected DCs cocultured or not with Vγ9Vδ2 T cells were added to naive CD4+ T cells. As shown in figure 5, the capacity of *Brucella*-infected DCs cocultured with Vγ9Vδ2 T cells to stimulate the proliferation of naive CD4+ T lymphocytes was increased when compared to *Brucella*-infected DCs alone, though still lower than that observed with *E. coli*-infected DCs (positive control). Moreover, to rule out that the effects observed were not caused by antigen-presenting activity of γδ T cells in the co-cultures, HMB-PP-activated γδ T cells were cocultured only with naive CD4+ T cells without the presence of DCs. In this condition, no or low proliferation of naive CD4+ T cells were observed as for DCs alone (infected or not). Altogether these results suggest that Vγ9Vδ2 T cells can induce antigen-presenting activity of *Brucella*-infected DCs and thus trigger the proliferation of naive CD4+ T lymphocytes.

### DCs Already Infected by *Brucella* for 24 h Keep the Ability to Activate Vγ9Vδ2 T Cells

**Figure 6:**
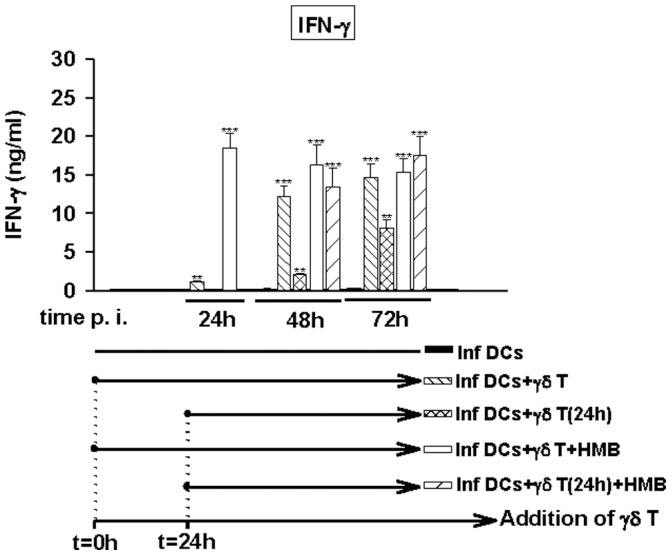
*Brucella*-infected DCs induce Vγ9Vδ2 T cells to produce IFN-γ even when they are added 24 h p.i. *Brucella*-infected DCs (MOI = 20) were cultured alone or in the presence of untreated or stimulated (0.2 nM HMB-PP) Vγ9Vδ2 T cells. Vγ9Vδ2 T cells were added to DCs 1 h or 24 h p.i. At 24 h, 48 h and 72 h p.i., supernatants were collected. IFN-γ was assessed by ELISA test. Data shown are the mean +/− SD of triplicates and representative of three independent experiments performed with cells from different donor. A significant difference between infected DCs in the presence or not of Vγ9Vδ2 T cells was calculated by using Student’s t test and is indicated by (**) where p<0.01 and (***) where p<0.001.

In the prospects to use Vγ9Vδ2 T cells as an adjuvant to help and/or trigger the maturation and presentation activity of DCs infected by pathogens impairing these processes, Vγ9Vδ2 cells were added either immediately after infection (as above) or after a delay (24 h p.i). Then, we studied the ability of Vγ9Vδ2 T cells to be activated by infected DCs in both conditions and then to restore the maturation and the presentation activity of DCs. In Fig. 6, we analysed the activation state of Vγ9Vδ2 T cells by measuring the production of IFN-γ. In the presence of HMB-PP, we observed a rapid and sustained production of IFN-γ, 24 h after the addition of Vγ9Vδ2 T cells. However, when Vγ9Vδ2 T cells are activated by the presence of infected DCs (and not by HMB-PP), IFN-γ was produced with a delay. IFN-γ production was observed at t = 48 h p.i. when Vγ9Vδ2 T cells are added t = 0 p.i. (Fig. 6, plain grey bars) and at t = 72 h p.i when Vγ9Vδ2 T cells are added at 24 h p.i. (Fig. 6, grey striped bars). But no difference in the activation (kinetic and amplitude) is observed between Vγ9Vδ2 T cells added immediately after infection or after a 24 h delay (Fig. 6, plain and striped white bars). Taken together, these results indicate that infected DCs keep the ability to activate Vγ9Vδ2 T cells even when the cells are not immediately cocultured together suggesting that *Brucella* infection does not inhibit the mechanism.

### Full Maturation of DCs into IL-12 Producing Cells with Presenting Activity after Delayed Addition of Vγ9Vδ2 T Cells

**Figure 7:**
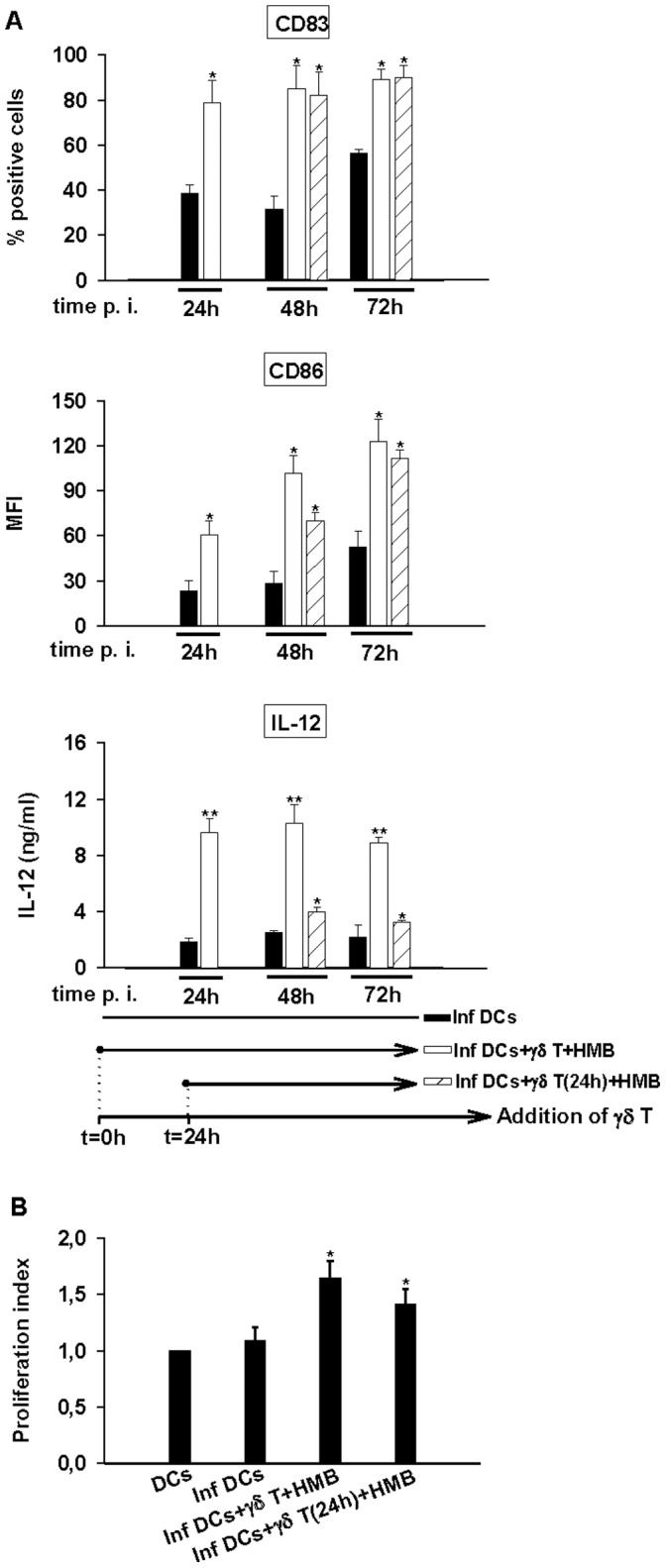
Activated Vγ9Vδ2 T cells are still able to induce maturation of *Brucella*-infected DCs even when they are added 24 h p.i. A/*Brucella*-infected DCs (MOI = 20) were cultured alone or in the presence of stimulated (0.2 nM HMB-PP) Vγ9Vδ2 T cells. Vγ9Vδ2 T cells were added to DCs 1 h or 24 h p.i. At 24 h, 48 h and 72 h p.i., supernatants were collected and cells were stained. CD83 and CD86 expression analysis were realized on CD1a+ cells. IL-12 was assessed by ELISA test in the collected supernatants. Data shown are the mean +/− SD of triplicates and representative of three independent experiments performed with cells from different donor. A significant difference between infected DCs in the presence or not of Vγ9Vδ2 T cells was calculated by using Student’s t test and is indicated by (*) where p<0. 05 and (**) where p<0.01. (B) *Brucella*-infected DCs (MOI = 20) were cultured alone or in the presence of stimulated (0.2 nM HMB-PP) Vγ9Vδ2 T cells. Vγ9Vδ2 T cells were added either 1 h or 24 h p.i. In all cases, CFSE-stained naive CD4+ T cells were added 24 h p.i. with DC/T cell ratio 0.1. After 5 days, CD4+ T cell proliferation was analyzed by flow cytometry and the proliferation index was calculated by comparison with non-infected cells. Data shown are the mean of duplicates +/− SD of three independent experiments performed with cells from different donors. Statistical difference is calculated by comparison with *Brucella*-infected DCs by using wilcoxon rank test and is indicated by (*) where p<0. 05.

The delay observed to produce IFN-γ and to activate Vγ9Vδ2 T cells did not allow studying DC maturation with a 72 h infection protocol and *in vitro* we could not keep infected cells in culture longer than 72 h p.i. Nevertheless, as HMB-PP-stimulated Vγ9Vδ2 T cells are activated rapidly (24 h) after contact with DCs (Fig. 6, white and white striped bars), we could use this activation protocol to study the effects of a delayed addition of Vγ9Vδ2 T cells on DC maturation and activity. The increase of maturation markers CD83 and CD86 was observed at 24 h when Vγ9Vδ2 T cells were added at t = 0 p.i. and at 48 h when Vγ9Vδ2 T cells were added at t = 24 h p.i. and reached at the similar levels (Fig. 7A). Although the amounts of IL-12 produced by *Brucella*-infected DCs were much lower when phosphoantigen-activated Vγ9Vδ2 T cells were added after 1 day (Fig. 7A) compared to t0 DC/γδ cocultures, they efficiently triggered naive CD4+ T cell responses (Fig. 7B). Overall, this suggests that exogenous phosphoantigen-activated Vγ9Vδ2 T cells could be used to restore the function of DCs during infection and thus modulate the immune response.

## Discussion

To date, the contribution of Vγ9Vδ2 T cells as effector cells of innate immunity is well recognized [11], and several studies have been investigating the possibility of exploiting their effector properties in infections [30] [25] and cancers [31], [32]. However, much less is known about the ability of Vγ9Vδ2 T cells to act as cellular adjuvants bridging innate and adaptive immunity. Although several reports have brought evidence that Vγ9Vδ2 T cells can modulate DC activity, no studies have been conducted with intracellular pathogens known to infect and abrogate DC functions yet [17], [18], [19]. Here, we have addressed the potential adjuvant role of Vγ9Vδ2 T cells in DC activity using a bacterial infection model characterized by multiple immune dysfunctions as notably the ability to impair DC maturation [13], [14]. Firstly, we report that in the presence of *Brucella*-infected DCs, Vγ9Vδ2 T cells are activated and produce INF-γ. The amount of IFN-γ is correlated with the number of infected DCs as shown by the data obtained with increasing MOIs (Fig. 1A). In addition, TNF-α is also produced and as for IFN-γ, the production was correlated with the number of infected cells (data not shown). We previously reported that Vγ9Vδ2 T cells impair the intracellular development of *Brucella* in macrophages by a combination of mechanisms notably by killing infected cells [25]. To analyse the maturation process of infected DCs, the infection protocol was optimised and the conditions for which the killing of infected DCs is not too efficient were determined. We performed infections with various ratios of Vγ9Vδ2 T cells/DCs (5∶1, 2∶1, 1∶1) and observed a high rate of infected DC death for the ratios of 5∶1 and 2∶1, not allowing to perform maturation process analyses (data not shown). Also, the use of higher concentrations of exogenous phosphoantigens (HMB-PP: 0.2 to 5 nM) leads to a powerful activation of Vγ9Vδ2 T cell cytotoxic activity (measured by CD107a staining, Fig. S4) which is correlated with a decrease of CD1a + cells in coculture (data not shown). From these data, we concluded that through their effector functions, Vγ9Vδ2 T cells impair the development of *Brucella* by killing infected DCs. Contrary to immature or mature non-infected DCs, *Brucella*-infected DCs trigger cytokine production and also cytolytic activity of Vγ9Vδ2 T cells. This means that the activation mechanisms involved are different and suggests that mechanisms specific to infected DCs trigger in Vγ9Vδ2 T cells intracellular signalling pathways which regulate their cytotoxic activity. To investigate the maturation process and in agreement with Meraviglia et al. and Devilder et al. reports and our own results, we performed infection experiments with a cell ratio of 1∶1 and with no or low HMB-PP (0.2 nM). These conditions are a good compromise between Vγ9Vδ2 T cells activation and the elimination of DCs and allow collecting enough DCs to analyse their phenotype and their function. Moreover, the physiological relevance of these experiments is not affected because it is likely that depending on the ratios used, the biological responses triggered by γδ T cells will be adapted to their environment (with high ratio killer/target cells, the cytotoxic activity will be more preponderant whereas with low ratios, other activities will be more active.

The adjuvant potential of Vγ9Vδ2 T cells resides in their ability to induce maturation of DCs into APCs that will promote the activation and differentiation of naive T cells into the appropriate class of effector cells. Phosphoantigen-activated Vγ9Vδ2 T cells are capable of driving maturation of iDC into APCs [17]. Here, we showed that not only exogenous phosphoantigen-activated Vγ9Vδ2 cells but also infected DC-activated Vγ9Vδ2 T cells have the capacity to trigger the maturation even when DCs are infected by pathogens which interfere and totally abrogate the maturation process. To go further in our investigations, we analysed the role played by contact-dependent mechanisms versus soluble factors. We demonstrated that a combination of both mechanisms is required to trigger a full maturation state and functional activity of *Brucella*-infected DCs. Among soluble factors, we investigated the role of IFN-γ and TNF-α which are produced following Vγ9Vδ2 T cell activation and showed their different contribution. TNF-α is involved in the up-modulation of CD83 and CD86 expression, IFN-γ in CD86 expression, in a lesser extent in CD83 and mainly responsible for IL-12 production. These results are consistent with the works of Ismaili et al. which showed an involvement of TNF-α and IFN-γ in maturation process of non-infected DCs and IL-12 production induced by phosphoantigen-activated Vγ9Vδ2 T cells [18] and also with those of Billards et al., which demonstrated an important role of TNF-α in the maturation process of *Brucella*-infected DCs while no or weak effect in the production of IL-12 [13]. Moreover, as the blockade of both TNF-α and IFN-γ did not totally abrogate the effects of Vγ9Vδ2 T cells, we can conclude that other mechanisms intervene in the maturation process of DCs in IL-12-producing cells (Fig. 4). Also, we observed in the experiments with trans-well systems that the effects of HMB-PP-activated Vγ9Vδ2 T cells are not totally abrogated particularly the production of IL-12 (Fig. 3). This suggests that cell-cell contact could be required for full maturation process and production of IL-12. In several studies, CD40L/CD40 interaction has been reported as implicated in iDCs maturation [33], [34], we performed the infection experiments in the presence of a blocking anti-CD40L mAb, and no effect has been observed suggesting that other membrane-bound molecules have to be involved (data not shown).

We could also demonstrate that not only activated Vγ9Vδ2 T cells have the ability to induce the maturation process of *Brucella*-infected DCs in IL-12-producing cells but also present APC properties and activate naive T cells proliferation. This confirms that Vγ9Vδ2 T cells can be used as an adjuvant for DC-based vaccines in infectious diseases. Moreover, several reports demonstrated that phosphoantigen-activated Vγ9Vδ2 T cells induce the maturation of DCs into APCs that release IL-12 but no or little amount of IL-10. Further investigations demonstrated that IFN-γ produced by Vγ9Vδ2 T cells is not sufficient to induce DC maturation [19], [35] but skews it towards Th1-inducing APCs [19]. Moreover, Dunne et al. also showed that phosphoantigen-activated Vγ9Vδ2 T cells strongly costimulate IL-12 production by DCs in response to LPS of *E. coli* and decrease IL-10 [19]. To determine the role of Vγ9Vδ2 T cells in the context of *Brucella* infection, we have measured IL-10 in supernatants. The infection of DCs by *Brucella* triggers neither IL-12 nor IL-10 production whereas *E. coli* LPS treatment triggers both IL-12 and IL-10 production. In the presence of Vγ9Vδ2 T cells, IL-12 production (figure 7) is detected but no IL-10 even when the addition of Vγ9Vδ2 T cells is delayed of 24 h in the presence of HMB-PP (Fig. S5). This suggests that, under conditions of bacterial infection, where LPS is present, activated Vγ9Vδ2 T cells induce full maturation of DCs into APC that release large amounts of IL-12, no or low amount of IL-10 and promote Th1 response. In this context, IL-10 cannot be a factor impairing the adjuvant role of γδ T cells.

In addition, it has been reported that in some bacterial infection, infected DCs partially activate naive Vγ9Vδ2 T cells. Mycobacterium tuberculosis-infected DCs induce proliferative, but not cytokine and cytolytic responses of Vγ9Vδ2 T cells [36]. This ineffective activation of Vγ9Vδ2 T cells is due to a deficient production of IL-15 by infected DCs and a blockage of differentiation of Vγ9Vδ2 T cells in a central memory stage where cells did not exhibit immediate effector functions [36]. On the contrary, we showed that IL-15 is produced by *Brucella*-infected DCs (data not shown) suggesting that Vγ9Vδ2 T cells might be fully activated and properly differentiated in effector memory cells in vivo.

Moreover, we have demonstrated that the bidirectional activating interaction between infected DCs and Vγ9Vδ2 T cells is effective when DC/γδ T cocultures are mixed immediately p.i. or after a delay and leading to a full functional APC activity of *Brucella*-infected DC.

In conclusion, our results and those of others provide evidence that Vγ9Vδ2 T cells are central players in immune responses during infection. They can directly act as immediate effector cells and mediate direct cytotoxicity to infected cells but also influence the initiation of adaptive immune response through the recruitment and activation of other immune effector cells, promote maturation of local DCs into IL-12 producing and Th1-inducing APCs. This study provides evidences that Vγ9Vδ2 T cells might be used to modulate the outcome of infectious diseases by promoting an adjuvant effect in DC-based cellular therapies and vaccines.

## Supporting Information

Figure S1 **DCs infected or not with various MOI (2, 5, 20, and 50) of** ***Brucella*** **were cocultured with Vγ9Vδ2 T cells (ratio 1∶1).** At 48 h p.i., cells were harvested and stained with FITC-conjugated mAbs to CD83 or CD86. CD83 and CD86 expression analyses were realized on CD1a^+^ cells by flow cytometry. The values for the percentage of CD83+ DCs or the mean fluorescence intensity of CD86+ DCs are indicated in upper right corner of graphs. Data shown are the representative of three independent experiments. Each experiment was performed with cells from different donors.
(DOC)

Figure S2 ***Brucella-*** **infected DCs (MOI = 20) were cocultured or not with Vγ9Vδ2 T cells (ratio 1∶1).** For positive control of maturation, uninfected DCs were supplemented with 1 µg/ml *E.coli* LPS. At 48 h p.i., supernatants were collected and cells were stained with FITC-conjugated mAbs to CD83 (A), CD86 (B). CD83 and CD86 expression analyses were realized on CD1a^+^ cells by flow cytometry. Data shown are the mean +/− SD of three independent experiments. IL-12 was assessed by ELISA (C). Data shown are the mean +/− SD of triplicates and are representative of three independent experiments. Significant differences between infected DCs and the other two groups was calculated by using Student’s *t* test (*p<0.05, **p<0.01). The difference between infected DCs in the presence of Vγ9Vδ2 T cells and uninfected DCs plus LPS was calculated by using Student’s *t* test but not statistically significant.
(DOC)

Figure S3 **DCs infected or not with** ***Brucella*** **were cocultured with primary Vγ9Vδ2 T cells (ratio 1∶1).** (A) Cells were harvested and stained with FITC-conjugated mAbs to CD83 or CD86. CD83 and CD86 expression analyses were realized on CD1a^+^ cells by flow cytometry. The values for the percentage and the mean fluorescence intensity of CD86+ DCs are indicated in upper right corner of graphs. Data shown are the representative of three independent experiments. Each experiment was performed with cells from different donors. (B) At 48 h p.i., supernatants were collected Data shown are the mean +/− SD of triplicates and are representative of three independent experiments. Significant differences between infected DCs alone and infected DCsthe in the presence of Vγ9Vδ2 T cells was calculated by using Student’s *t* test (**p<0.01).
(DOC)

Figure S4 **Expression of CD107a by Vγ9Vδ2 T cells.** Untreated or stimulated (0.2 nM or 5 nM HMBpp) Vγ9Vδ2 T cells were cocultured with non- or *Brucella*-infected DCs (MOI = 20) for 24 h with a ratio 1∶1 DCs/Vγ9Vδ2 T cells. PE-conjugated anti-CD107a mAb and monensin were added 5 h and 4 h respectively before cell harvesting. At 24 h p.i., FITC-conjugated γ9 mAb or its isotype-matched control were incubated for 30 min at 4°C with harvested cells. CD107a expression analyses were realized on γ9+ cells by flow cytometry and the results were expressed as percentage of positive cells and indicated directly on the graph.
(DOC)

Figure S5 **Measurement of IL-10 in supernatants of infection.** *Brucella*-infected DCs (MOI = 20) were cultured alone or in the presence of stimulated (0.2 nM HMB-PP) Vγ9Vδ2 T cells. Vγ9Vδ2 T cells were added to DCs 1 h or 24 h p.i. At 24 h, 48 h and 72 h p.i., supernatants were collected and IL-10 was assessed by ELISA test. Data shown are the mean +/− SD of triplicates and representative of three independent experiments performed with cells from different donor. A significant difference between infected DCs in the presence or not of LPS was calculated by using Student’s t test and is indicated by (**) where p<0.01.
(DOC)
