# Supplementary material for: Full Restoration of Brucella-Infected Dendritic Cell Functionality through Vγ9Vδ2 T Helper Type 1 Crosstalk
Source: PLoS One. 2012 Aug 22;7(8):e43613. doi: 10.1371/journal.pone.0043613 (PMC3425473; doi:10.1371/journal.pone.0043613)
Supplement: Figure S1 — DCs infected or not with various MOI (2, 5, 20, and 50) of Brucella were cocultured with Vγ9Vδ2 T cells (ratio 1∶1). At 48 h p.i., cells were harvested and stained with FITC-conjugated mAbs to CD83 or CD86. CD83 and CD86 expression analyses were realized on CD1a+ cells by flow cytometry. The values for the percentage of CD83+ DCs or the mean fluorescence intensity of CD86+ DCs are indicated in upper right corner of graphs. Data shown are the representative of three independent experiments. Each experiment was performed with cells from different donors. (DOC) [file pone.0043613.s001.doc]

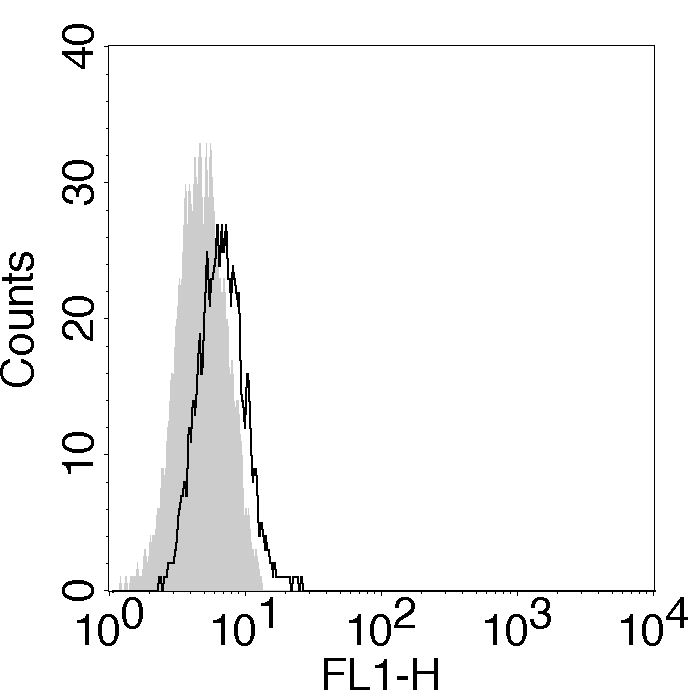

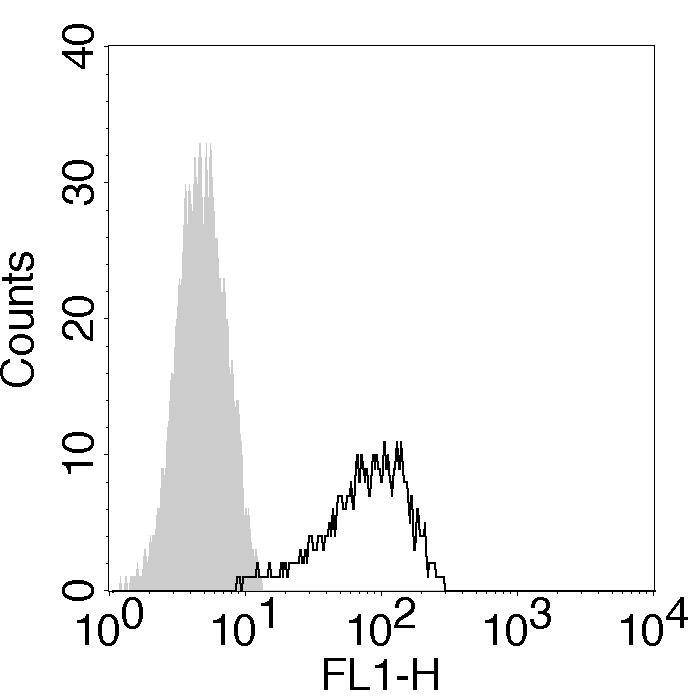

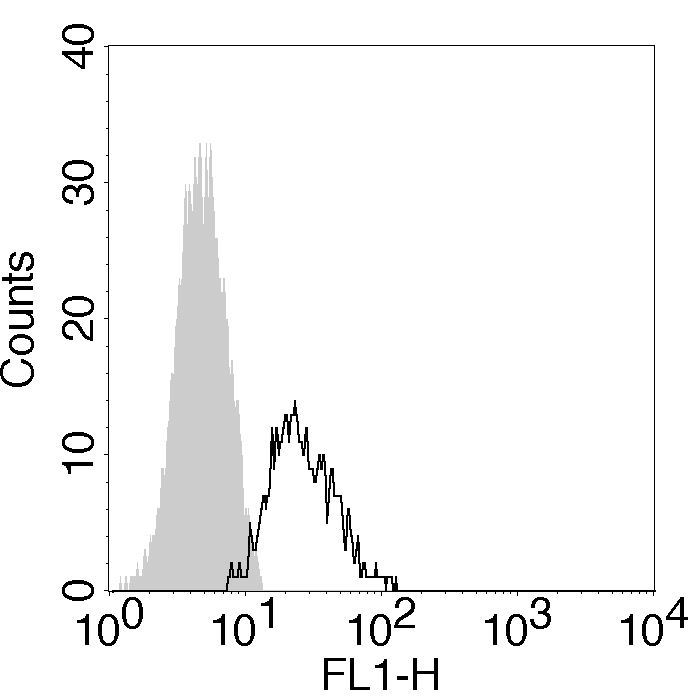

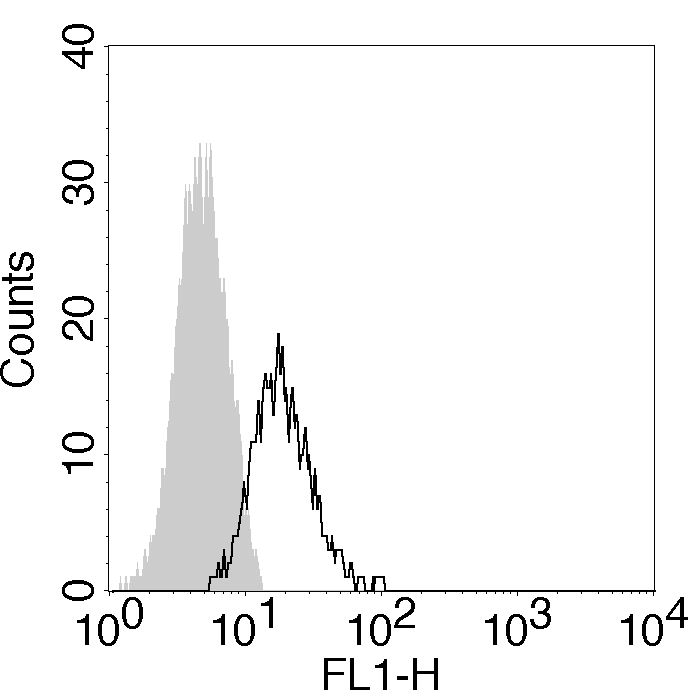

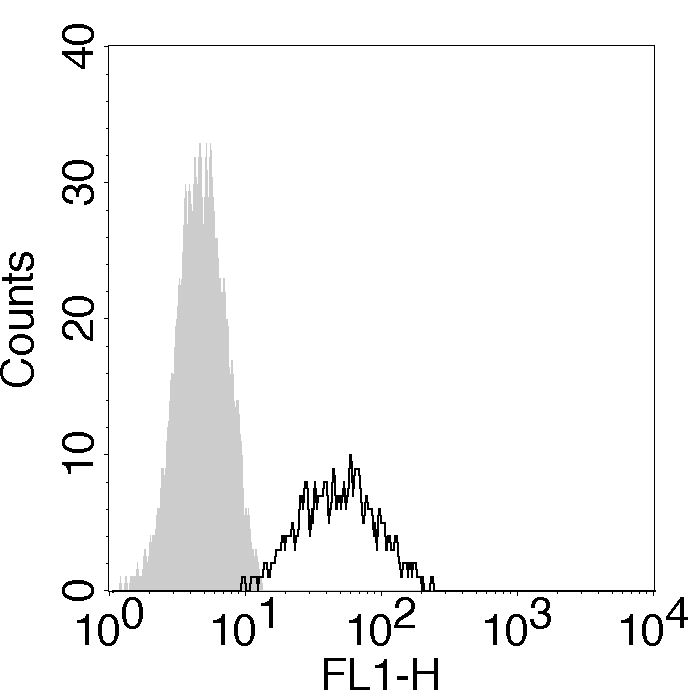

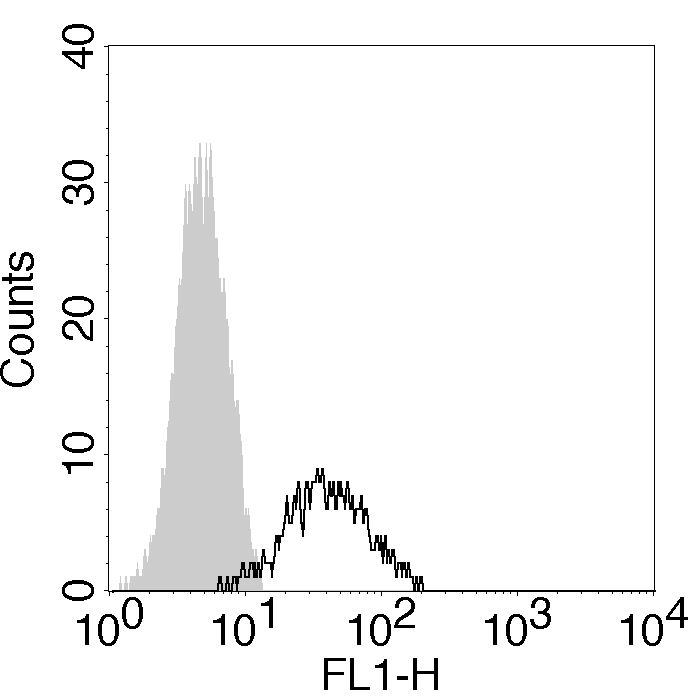

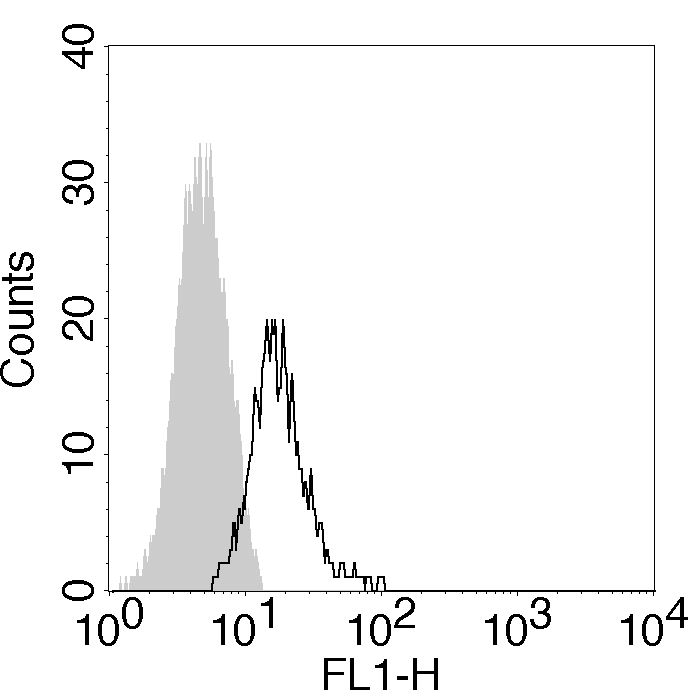

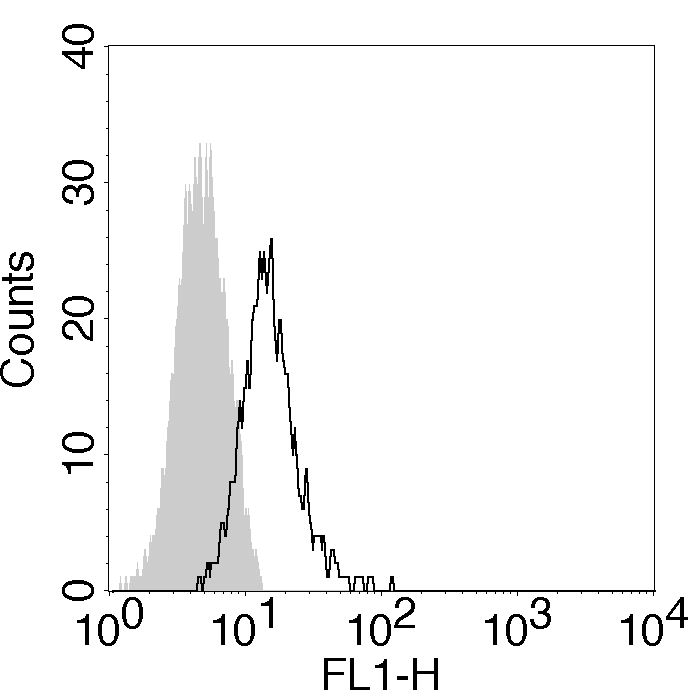

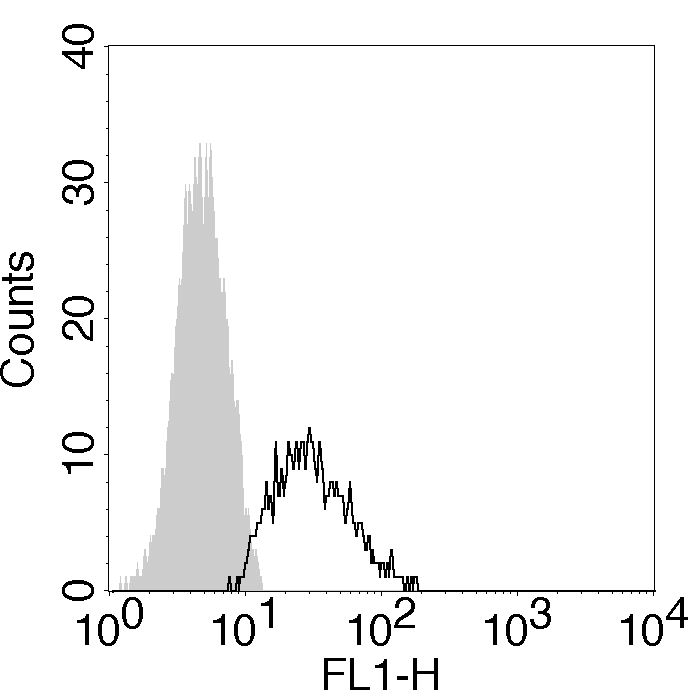

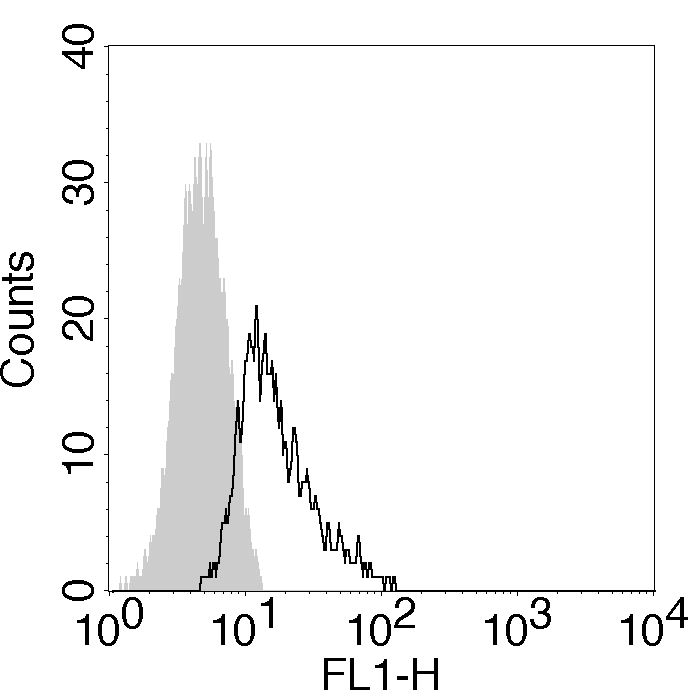

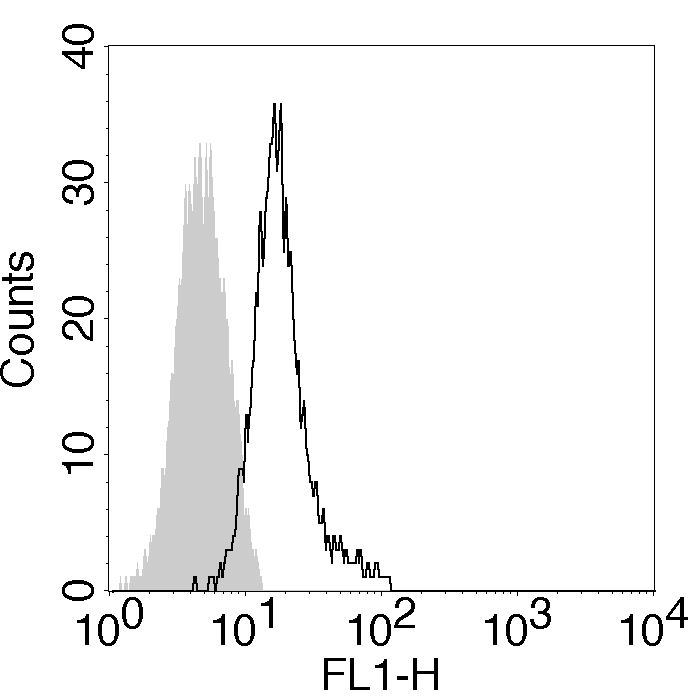

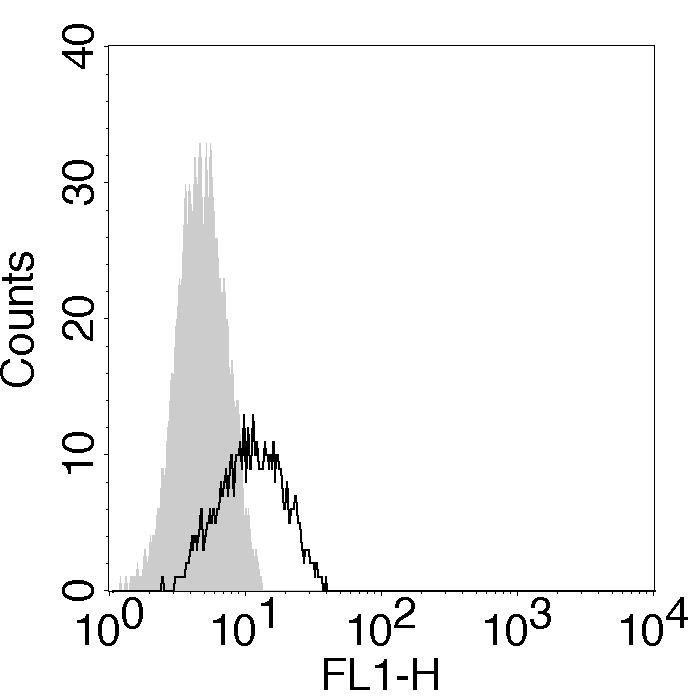

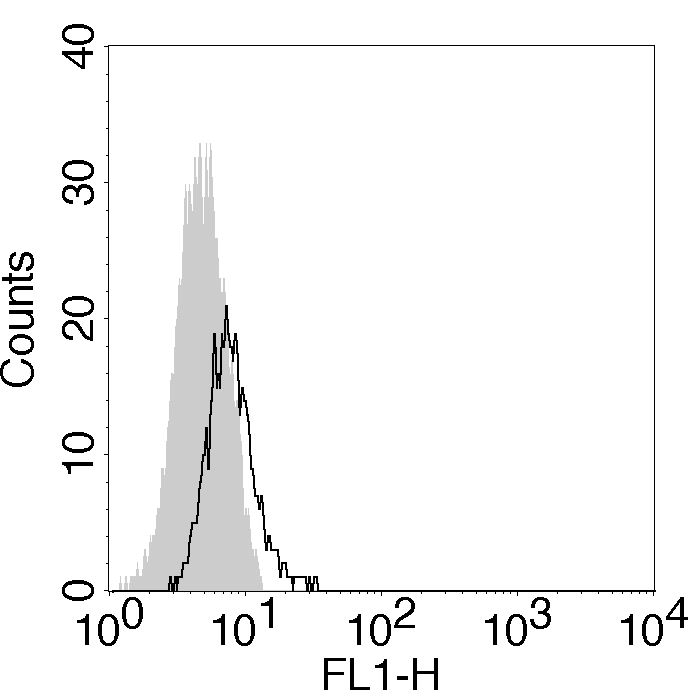

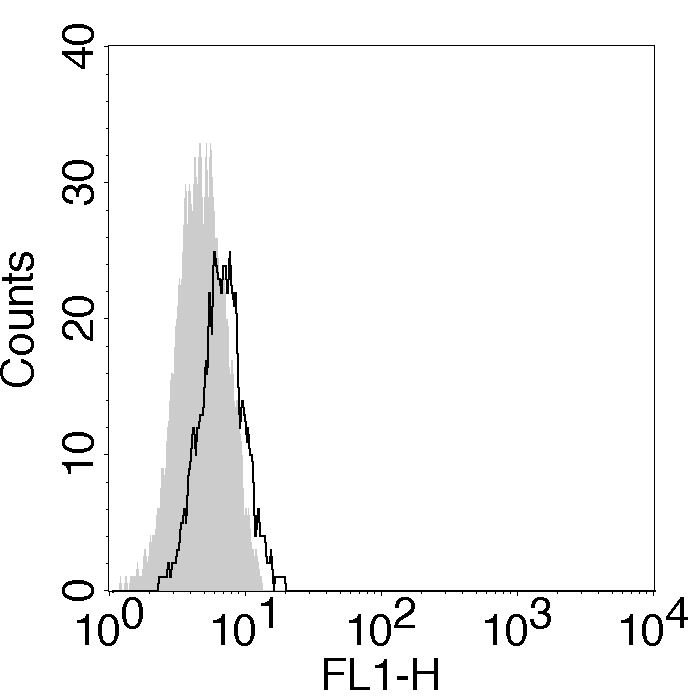

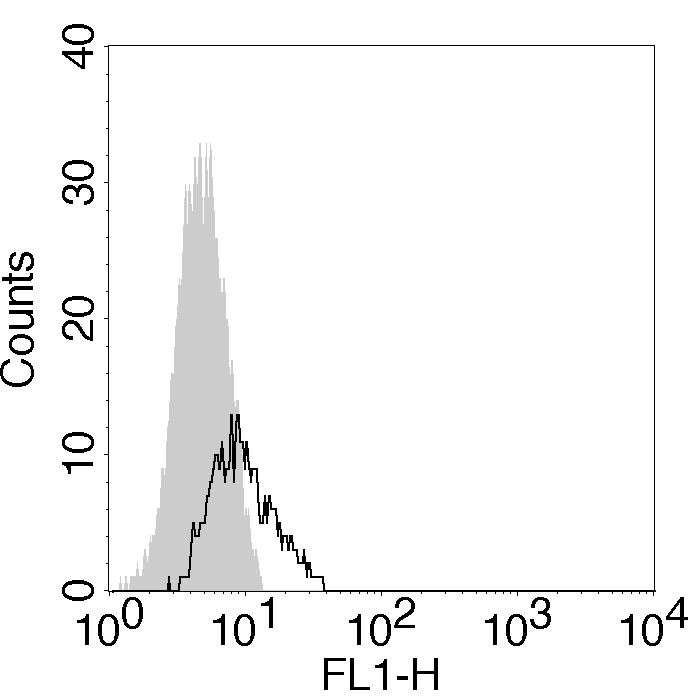

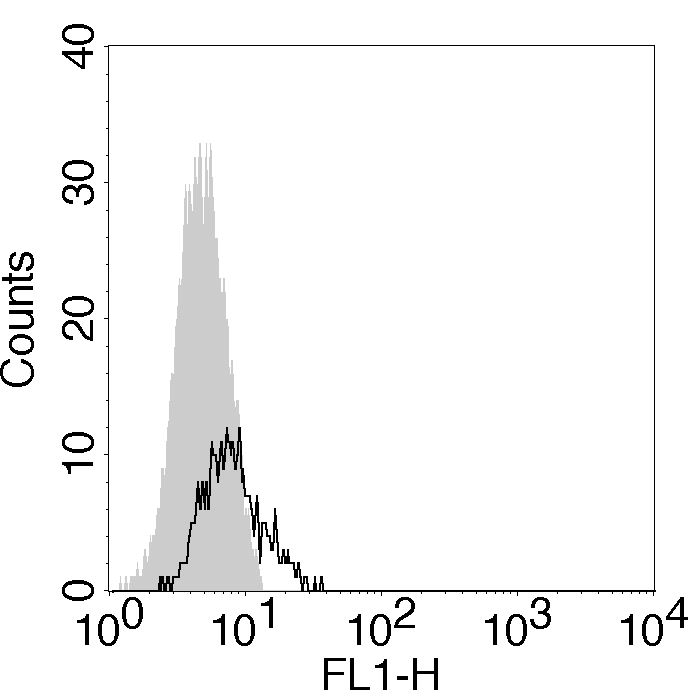

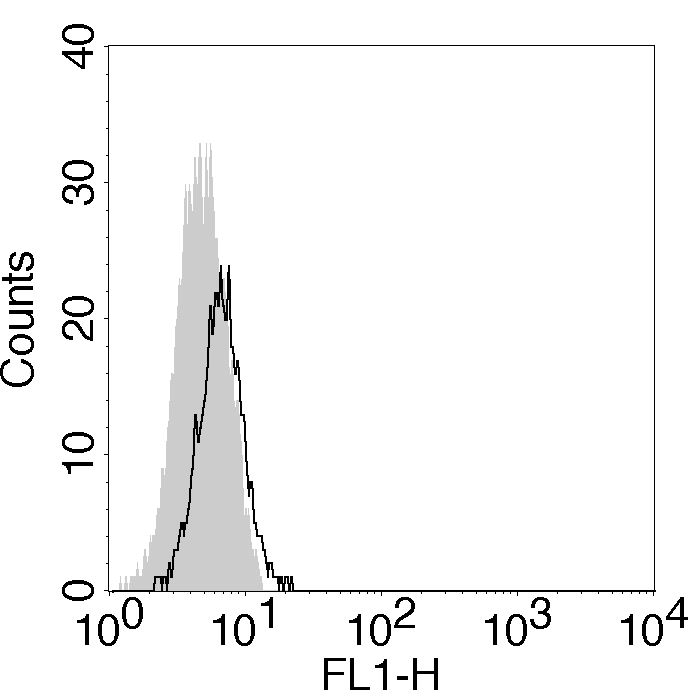

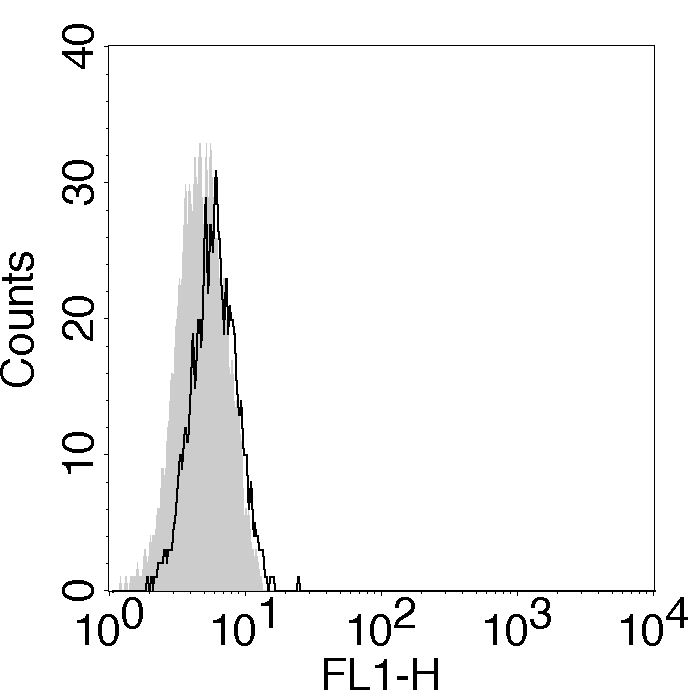

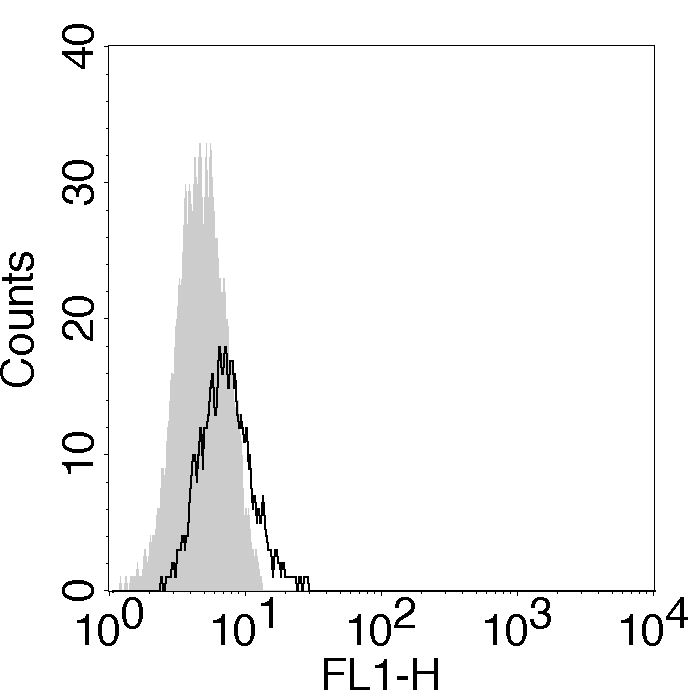

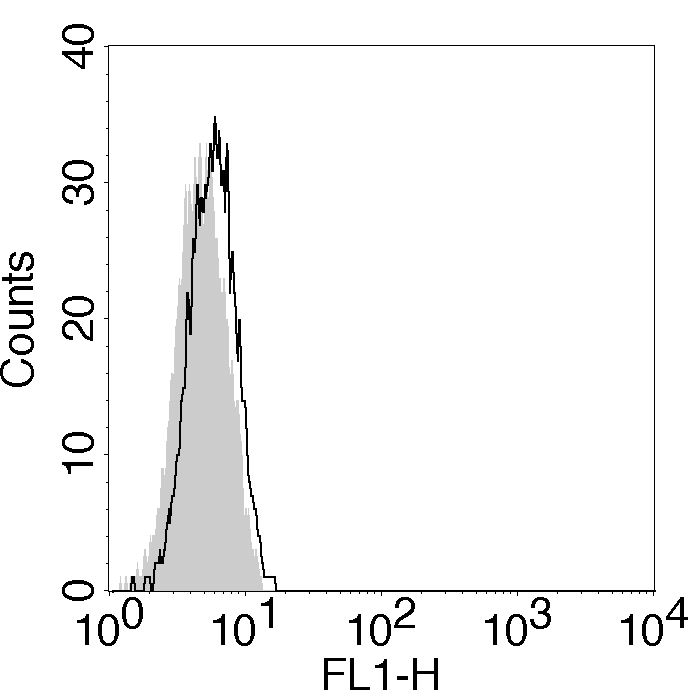


**Counts**

**Counts**

**0 bact**

**2 bact**

**5 bact**

**20 bact**

**50 bact**

**DCs**

**DCs+γδ T**

**DCs**

**DCs+γδ T**

9.9%

4.7%

26.7%

9.3%

37.5%

15.3%

47.5%

25.1%

62.2%

MFI=21.2

MFI=16.1

MFI=38.8

MFI=18.4

MFI=51.2

MFI=20.4

MFI=60.6

MFI=29.9

MFI=85.5

**CD83**

**CD86**

**Isotype control**

**DCs +/- γδ T**

12.6%

MFI=20.5
