# Supplementary material for: Full Restoration of Brucella-Infected Dendritic Cell Functionality through Vγ9Vδ2 T Helper Type 1 Crosstalk
Source: PLoS One. 2012 Aug 22;7(8):e43613. doi: 10.1371/journal.pone.0043613 (PMC3425473; doi:10.1371/journal.pone.0043613)
Supplement: Figure S3 — DCs infected or not with Brucella were cocultured with primary Vγ9Vδ2 T cells (ratio 1∶1). (A) Cells were harvested and stained with FITC-conjugated mAbs to CD83 or CD86. CD83 and CD86 expression analyses were realized on CD1a+ cells by flow cytometry. The values for the percentage and the mean fluorescence intensity of CD86+ DCs are indicated in upper right corner of graphs. Data shown are the representative of three independent experiments. Each experiment was performed with cells from different donors. (B) At 48 h p.i., supernatants were collected Data shown are the mean +/− SD of triplicates and are representative of three independent experiments. Significant differences between infected DCs alone and infected DCsthe in the presence of Vγ9Vδ2 T cells was calculated by using Student’s t test (**p<0.01). (DOC) [file pone.0043613.s003.doc]

**Counts**

**CD83**


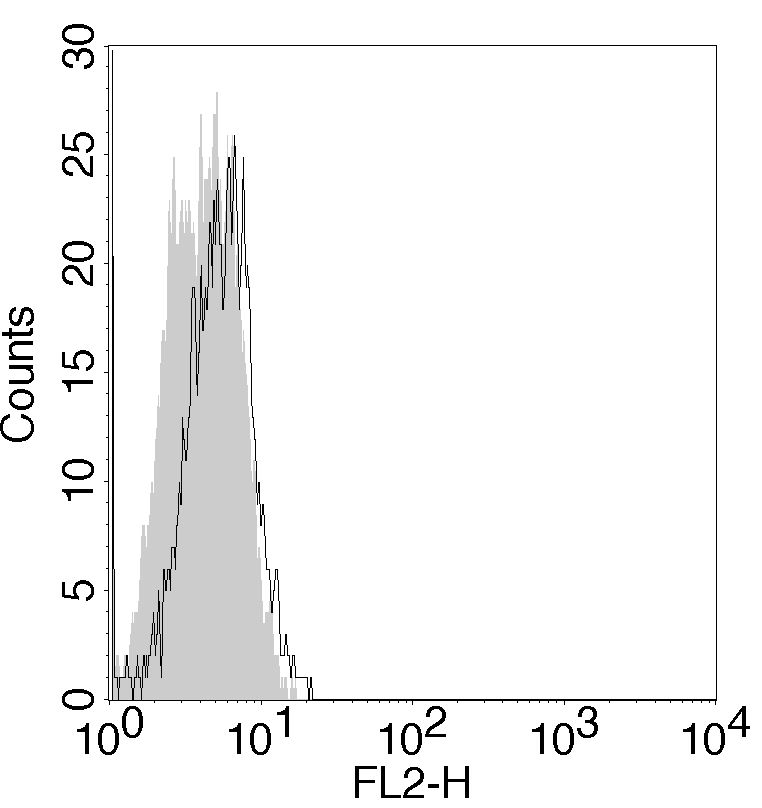

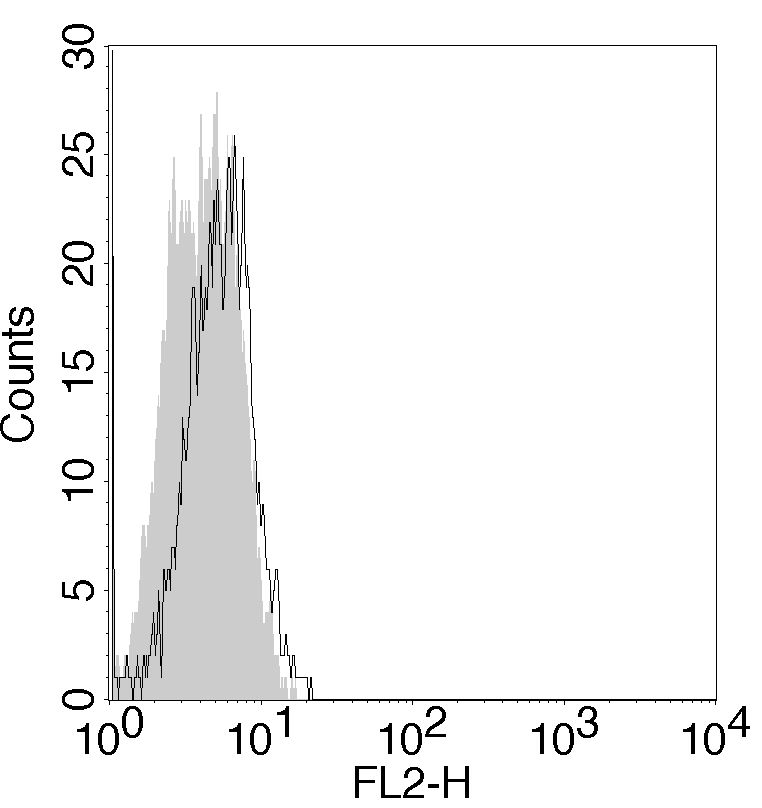

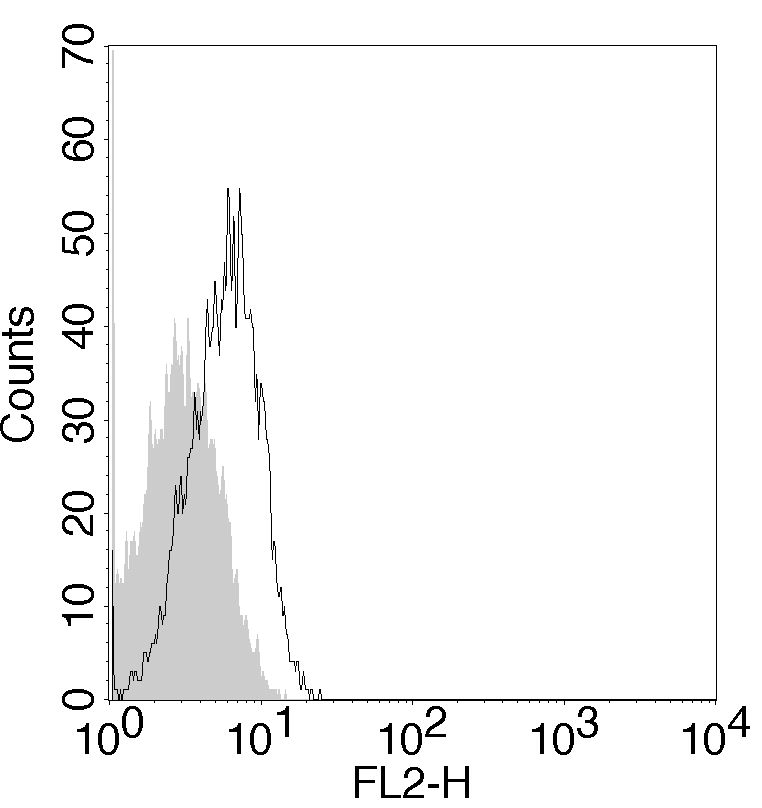

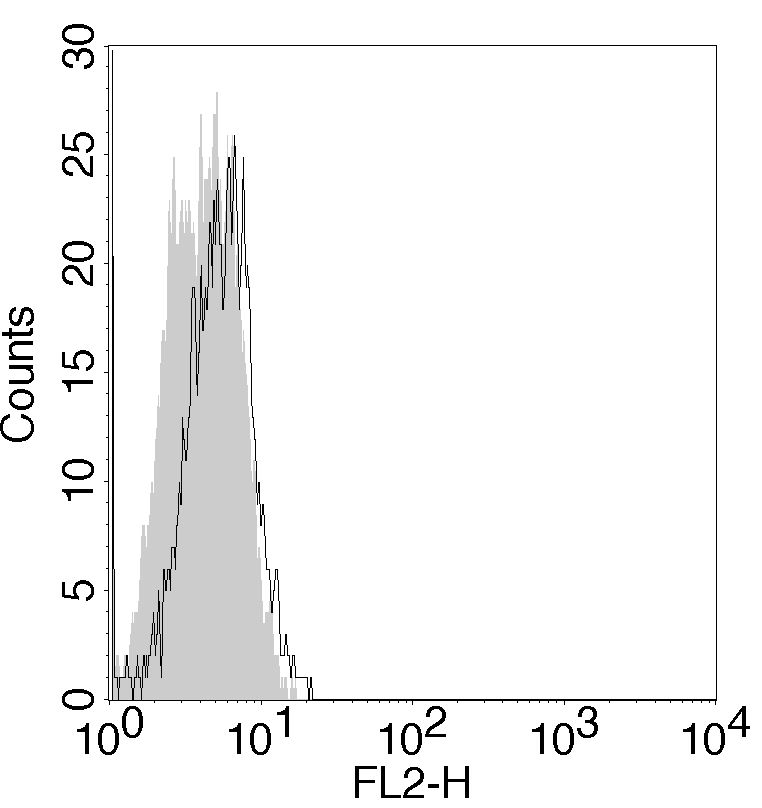

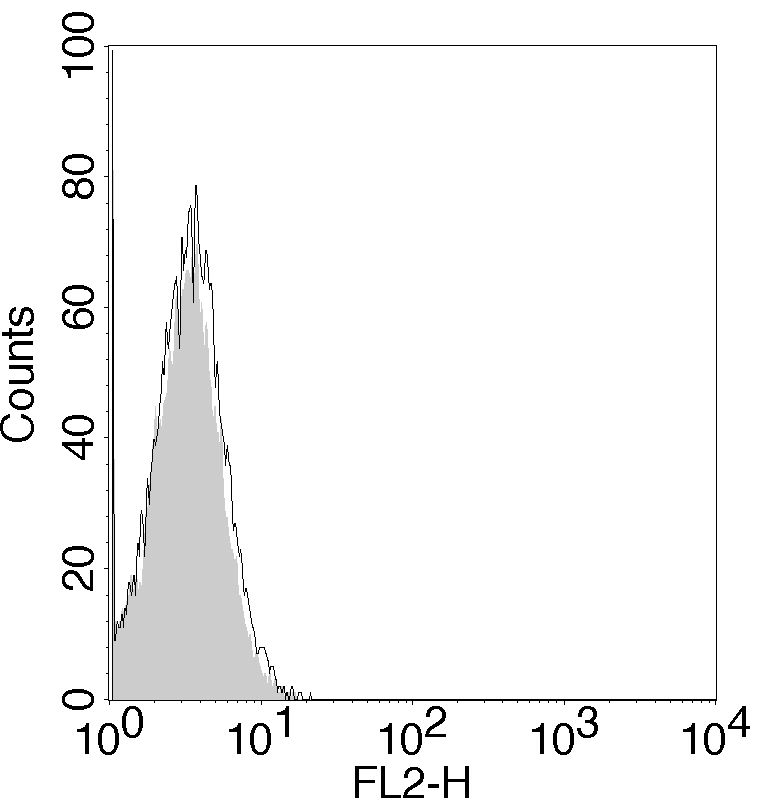

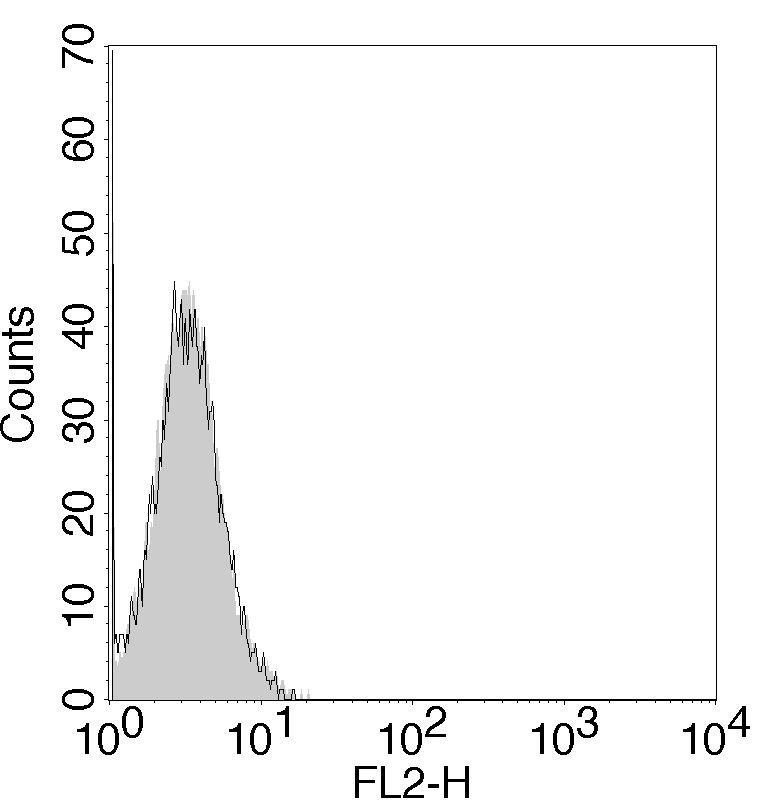

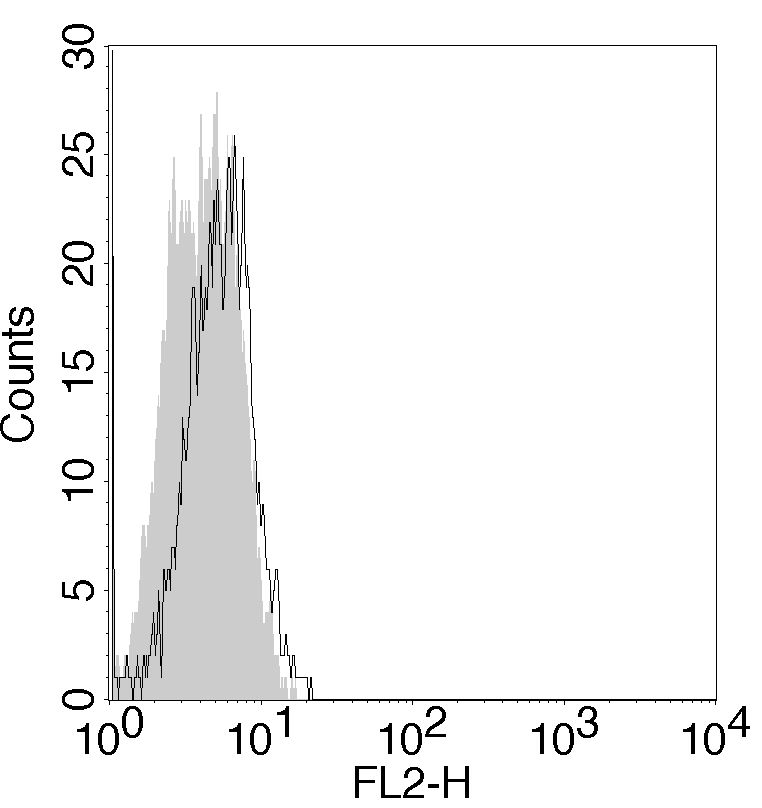


**DCs**

**DCs+γδ T**

20

40

60

0

20

40

60


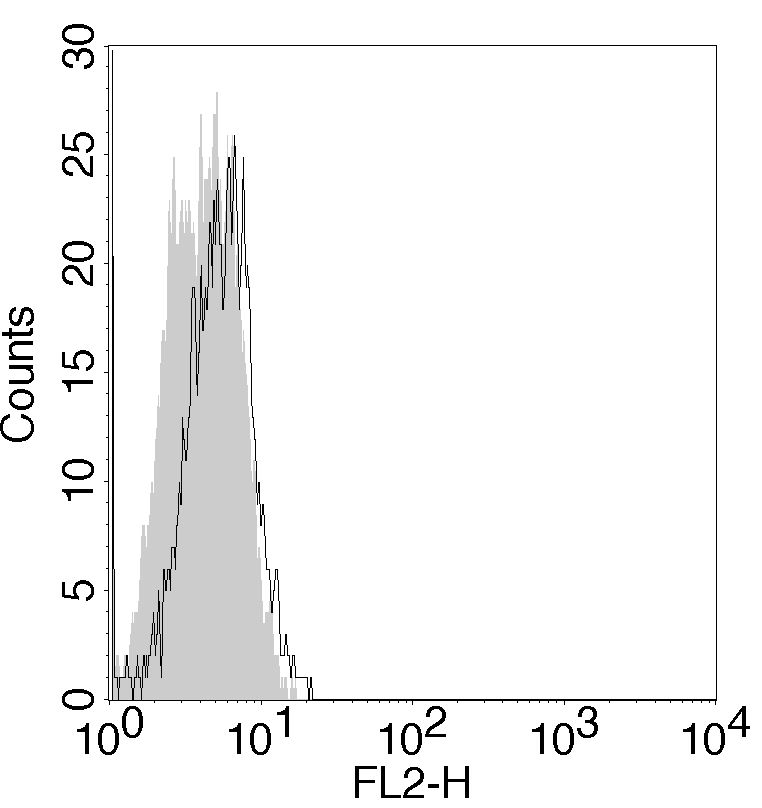


0

20

40

60

0

20

40

60

0

**DCs**

**DCs+γδ T**

**Counts**

**CD86**


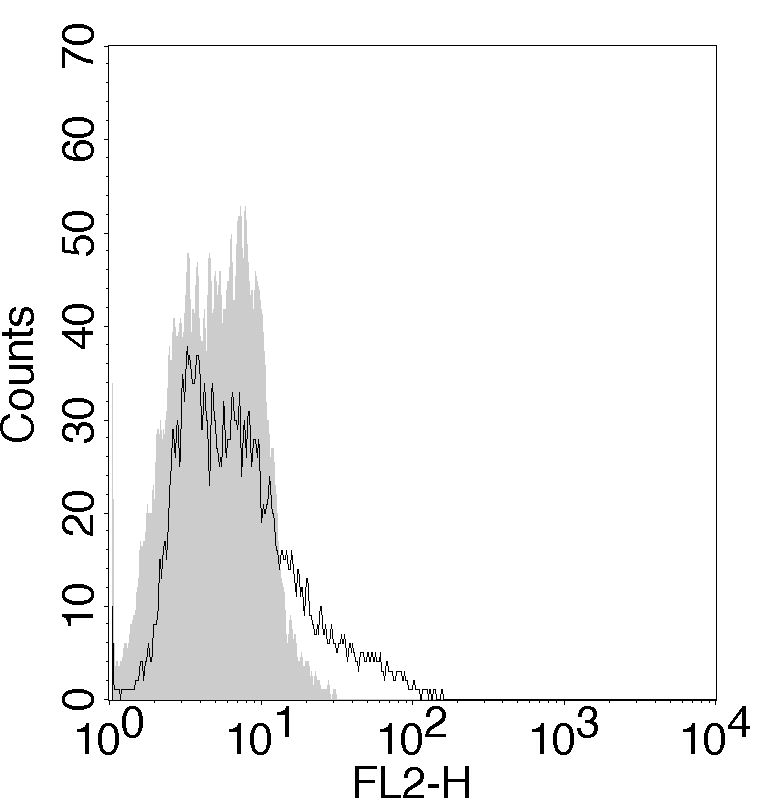

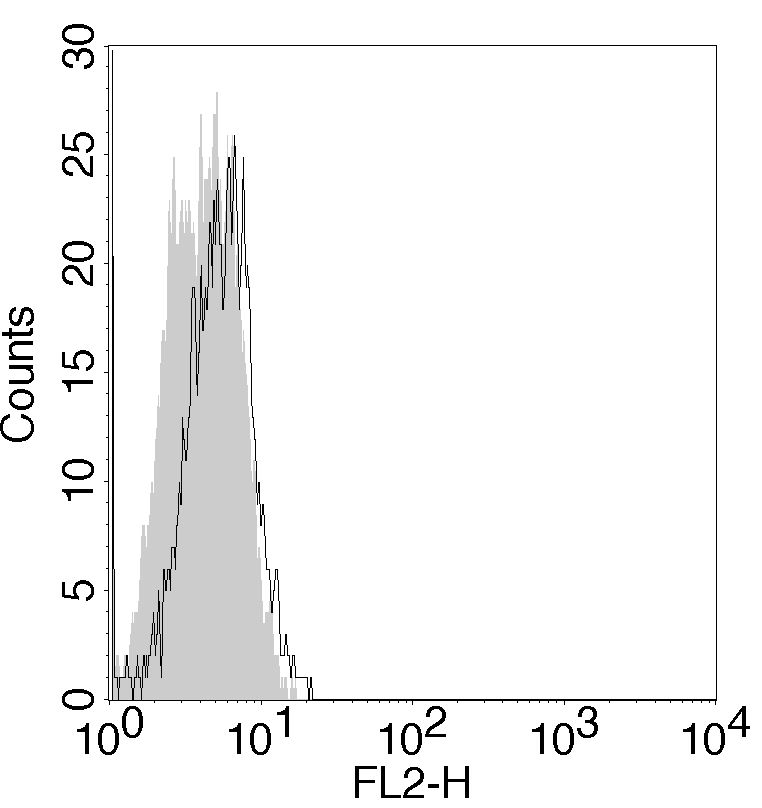

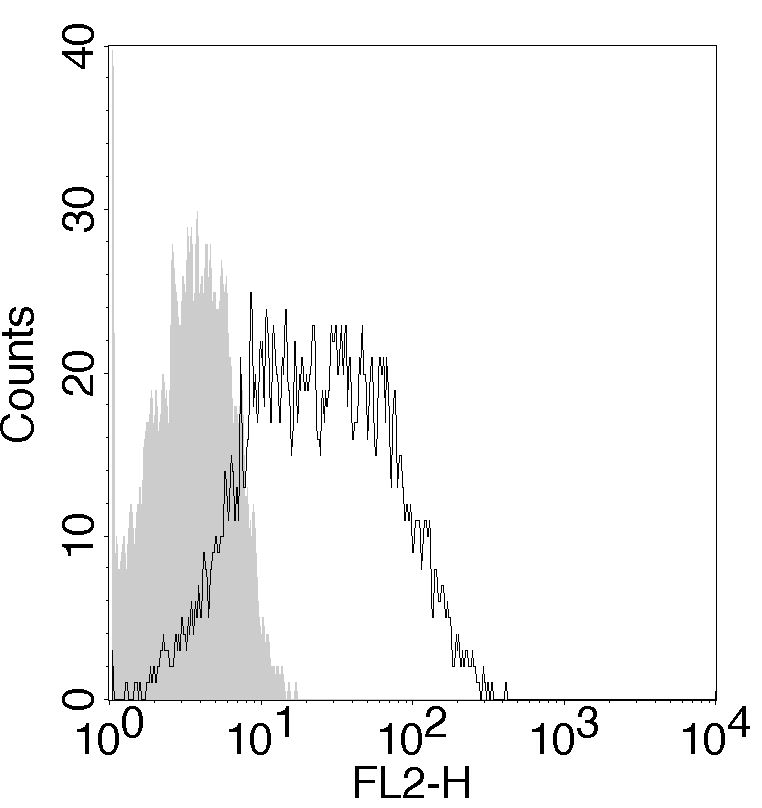

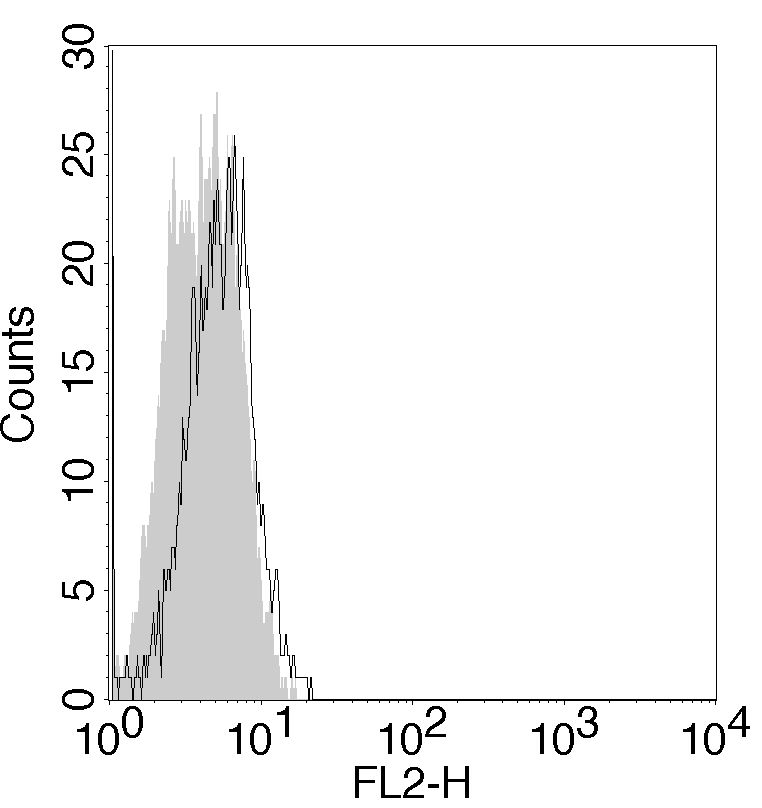

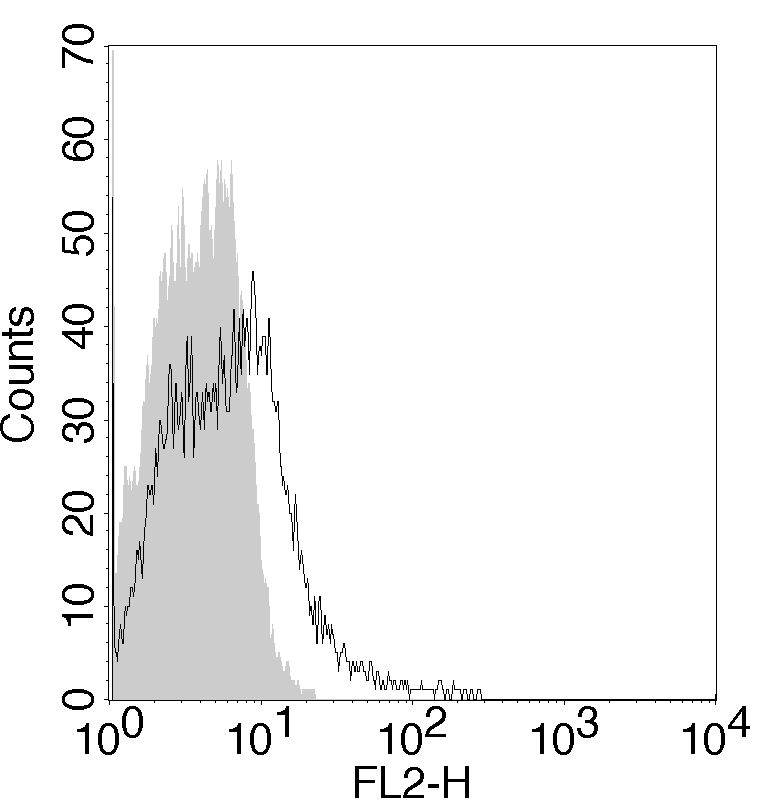

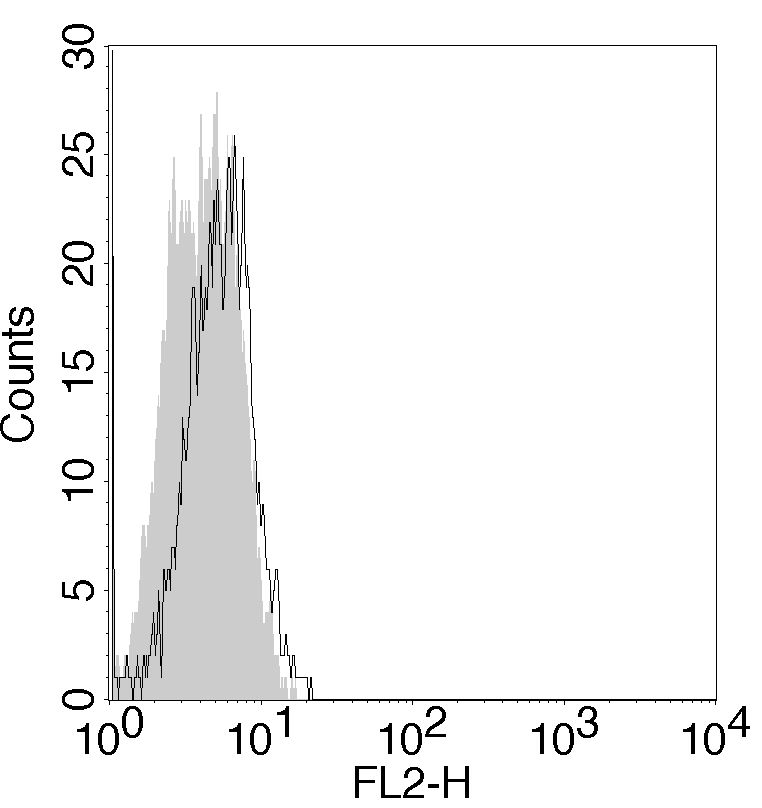

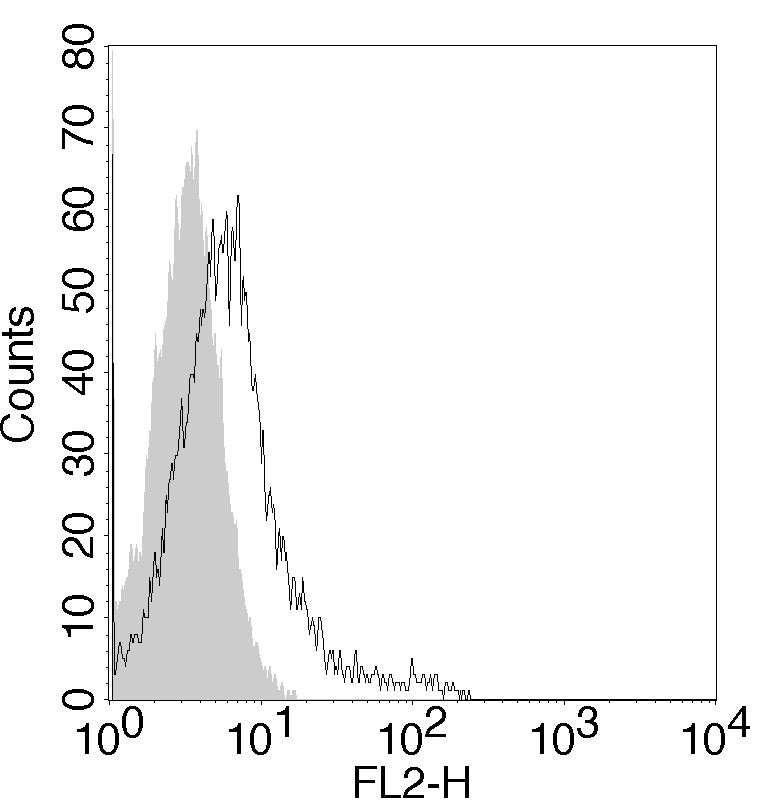

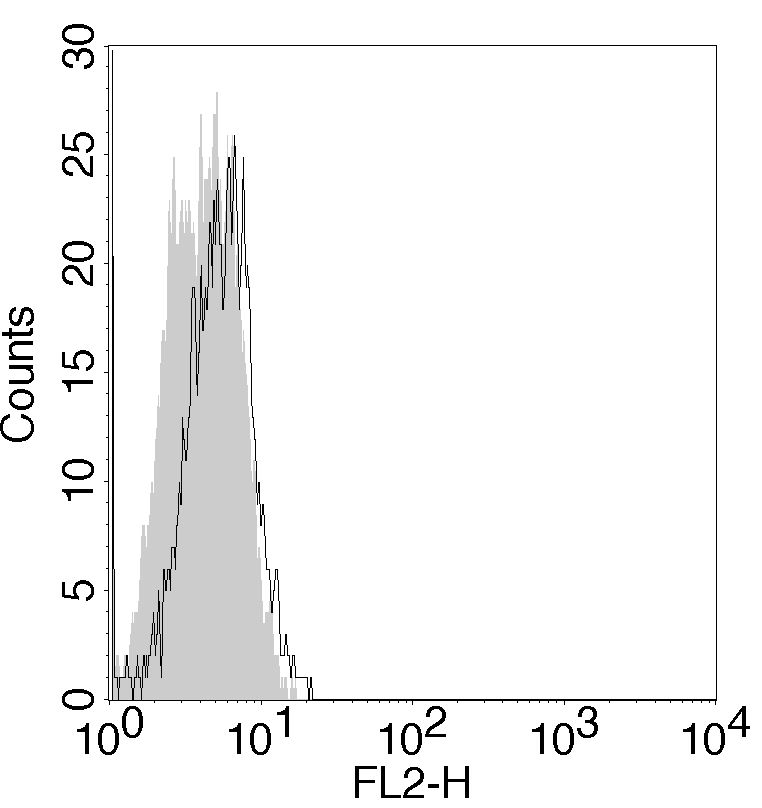


**0 bact**

**20 bact**

20

40

60

0

20

40

60

0

20

40

60

0

20

40

60

0

1.85%

MFI= 3.6

3%

MFI= 3.8

2.84%

MFI= 5.3

57.59%

MFI= 6.2

22.90%

MFI= 8.5

35.23%

MFI= 10.4

18.92%

MFI= 9.58

85.67%

MFI= 40.4

**A**

**B**

0

2

4

6

8

10

12


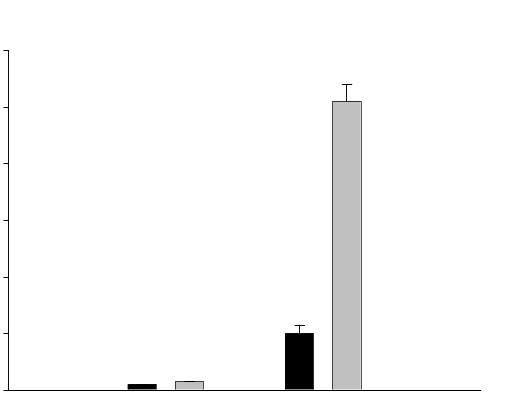


**Non INF**

**INF**

******

IL-12 (ng/ml)

**DCs**

**DCs + γδ T**

**DCs +/- γδ T**

**Isotype control**
