# Supplementary figures and images for: Full Restoration of Brucella-Infected Dendritic Cell Functionality through Vγ9Vδ2 T Helper Type 1 Crosstalk
Source: PLoS One. 2012 Aug 22;7(8):e43613. doi: 10.1371/journal.pone.0043613 (PMC3425473; doi:10.1371/journal.pone.0043613)

**CD107a**

**InfDCs+ T+HMB 5nM**

**InfDCs+ T+HMB 0.2nM**

**DCs+ T**

**Inf DCs+ T**

**FSC-H**


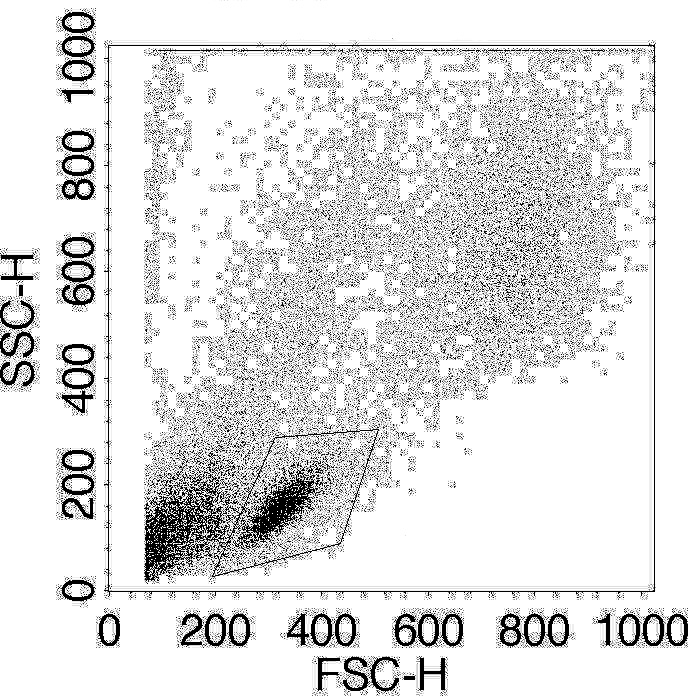

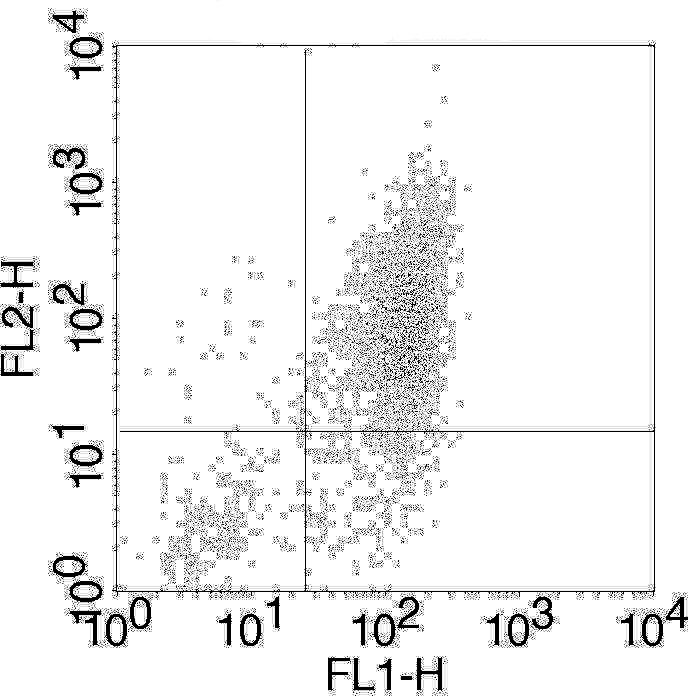


9.6%

80.0%


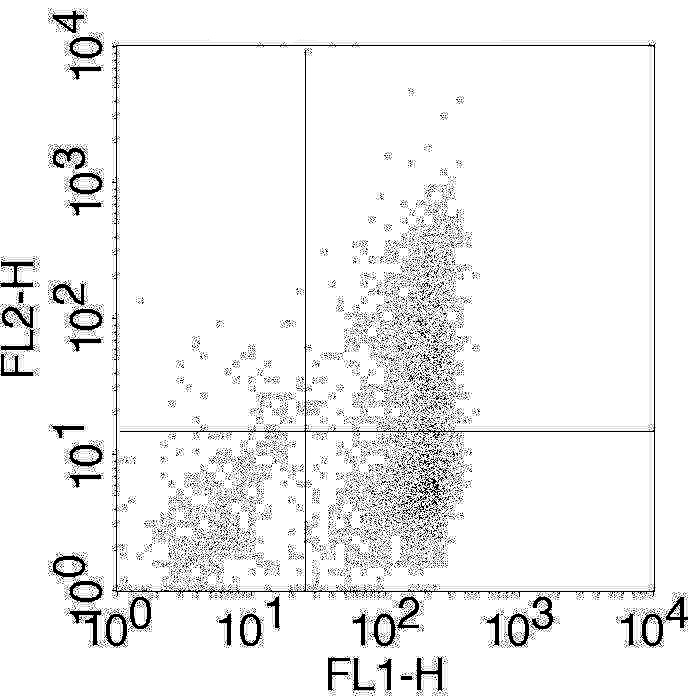


43.8%

40.4%


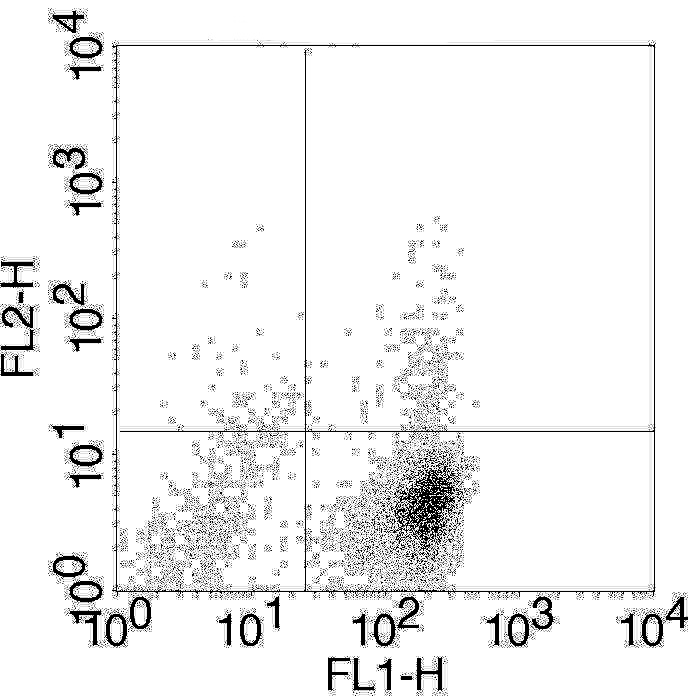


85.4%

3.8%


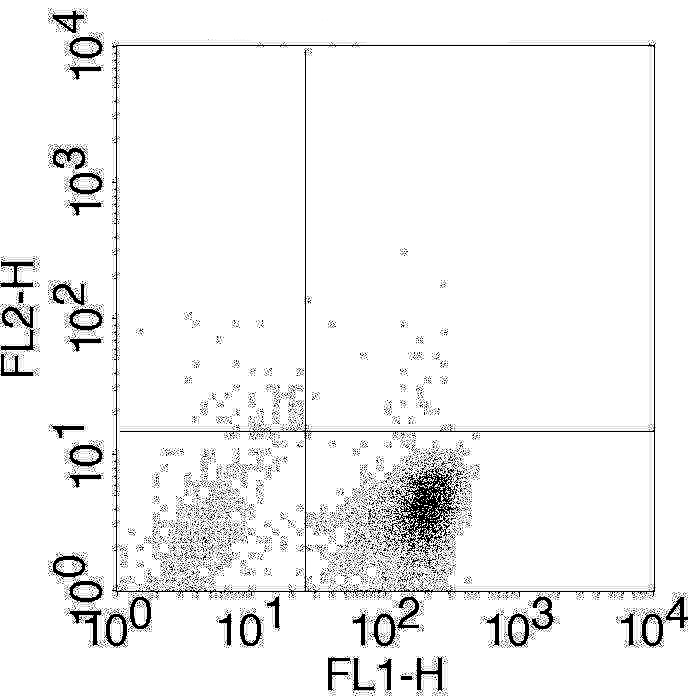


0.7%

87.7%

**γ9**

**SSC-H**

** T cells**

Supplement: Figure S4 — Expression of CD107a by Vγ9Vδ2 T cells. Untreated or stimulated (0.2 nM or 5 nM HMBpp) Vγ9Vδ2 T cells were cocultured with non- or Brucella-infected DCs (MOI = 20) for 24 h with a ratio 1∶1 DCs/Vγ9Vδ2 T cells. PE-conjugated anti-CD107a mAb and monensin were added 5 h and 4 h respectively before cell harvesting. At 24 h p.i., FITC-conjugated γ9 mAb or its isotype-matched control were incubated for 30 min at 4°C with harvested cells. CD107a expression analyses were realized on γ9+ cells by flow cytometry and the results were expressed as percentage of positive cells and indicated directly on the graph. (DOC) [file pone.0043613.s004.doc]
