# Supplementary material for: Full Restoration of Brucella-Infected Dendritic Cell Functionality through Vγ9Vδ2 T Helper Type 1 Crosstalk
Source: PLoS One. 2012 Aug 22;7(8):e43613. doi: 10.1371/journal.pone.0043613 (PMC3425473; doi:10.1371/journal.pone.0043613)
Supplement: Supplementary file 4 [file pone.0043613.s002.doc]

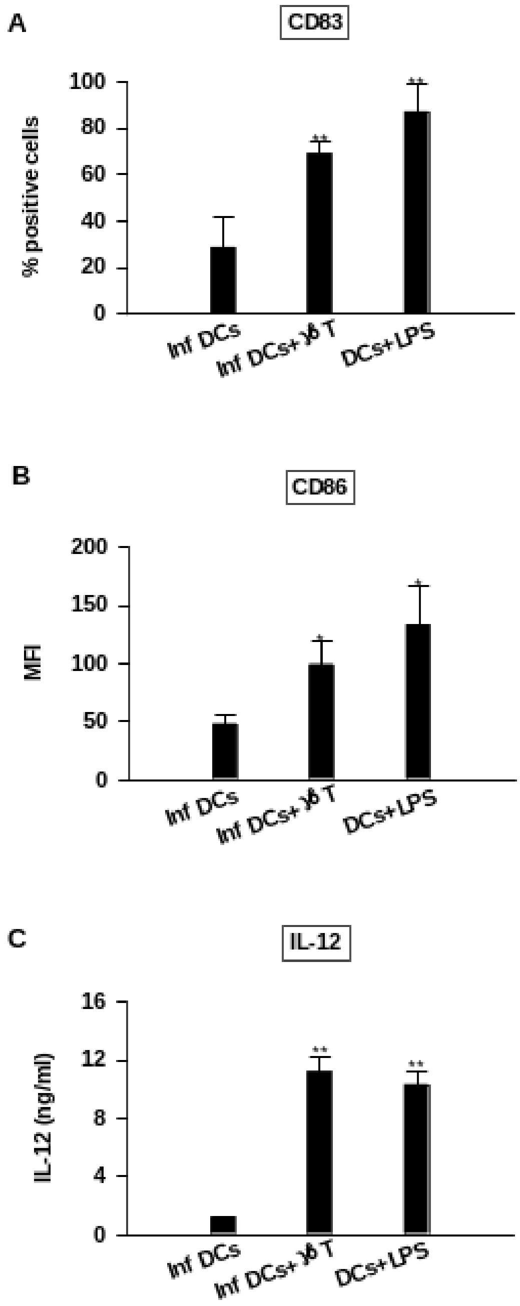
A
CD83
% positive cells
0
20
40
60
80
100
**
**
Inf DCs
Inf DCs+ γδ T
DCs+LPS
B
CD86
MFI
0
50
100
150
200
*
*
Inf DCs
Inf DCs+ γδ T
DCs+LPS
C
IL-12
IL-12 (ng/ml)
0
4
8
12
16
**
**
Inf DCs
Inf DCs+ γδ T
DCs+LPS
