# Supplementary material for: Full Restoration of Brucella-Infected Dendritic Cell Functionality through Vγ9Vδ2 T Helper Type 1 Crosstalk
Source: PLoS One. 2012 Aug 22;7(8):e43613. doi: 10.1371/journal.pone.0043613 (PMC3425473; doi:10.1371/journal.pone.0043613)
Supplement: Supplementary file 5 [file pone.0043613.s005.doc]

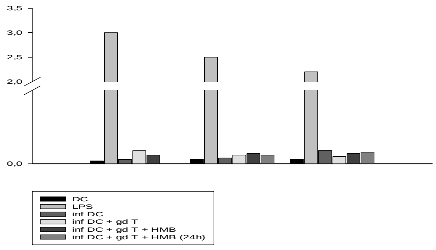
3,5
3,0
2,5
2,0
0,0
DC
LPS
inf DC
inf DC + gd T
inf DC + gd T + HMB
inf DC + gd T + HMB (24h)

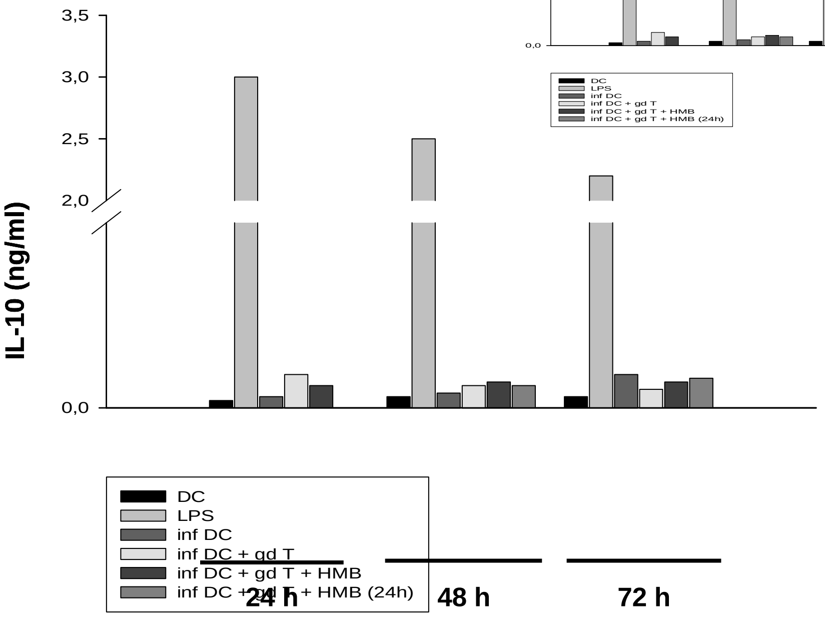
IL-10 (ng/ml)
3,5
3,0
2,5
2,0
0,0
DC
LPS
inf DC
inf DC + gd T
inf DC + gd T + HMB
inf DC + gd T + HMB (24h)
24 h
48 h
72 h
